# A rapid, inexpensive diagnostic for bacterial pathogen and resistance detection in resource-limited settings

**DOI:** 10.1126/sciadv.aeb6630

**Published:** 2026-10-02

**Authors:** David J. Roach, Bhrus Pun Sangruji, Shriya Bhat, Selama Tesfamariam, Ishay Ben-Zion, Miriam Bern, Josephine Bagnall, Noam Shoresh, Lisa Milien, Roby P. Bhattacharyya

**Affiliations:** ^1^ Division of Infectious Diseases, Brigham and Women’s Hospital, Boston, MA, USA.; ^2^ Infectious Disease and Microbiome Program, Broad Institute of MIT and Harvard, Boston, MA, USA.; ^3^ Tufts University, Boston, MA, USA.; ^4^ Harvard University, Boston, MA, USA.; ^5^ Kaiser Permanente Bernard J. Tyson School of Medicine, Pasadena, CA, USA.; ^6^ Department of Pathology, Massachusetts General Hospital, Boston, MA, USA.; ^7^ Division of Infectious Diseases, Department of Medicine, Massachusetts General Hospital, Boston, MA, USA.

## Abstract

Antimicrobial resistance is a major global health threat, with disproportionate impact in regions with limited diagnostic infrastructure. To address this challenge, we developed BADLOCK (Bacterial and AMR Detection by SHERLOCK), a rapid, low-cost molecular diagnostic platform for direct detection of bacterial pathogens and resistance genes from clinical samples. BADLOCK operates as a one-pot CRISPR-Cas13a reaction capable of detecting nine bacterial species and four major resistance genes directly from positive blood culture. It requires only a heat block and a basic fluorometer for operation. We validated BADLOCK on 194 sequentially collected clinical blood culture specimens growing Gram-negative rods, supplemented with 69 mock samples generated from banked isolates enriched for targeted resistance genes. In all, we conducted 2224 individual reactions, achieving 97.6% accuracy (2171 of 2224) at the reaction level. At the assay level, 89.5% (274 of 306) showed perfect or partial concordance with gold-standard species and resistance gene detection, including 255 assays with perfect concordance and 19 with partial concordance (correct detection of at least one pathogen). This included an evaluation of BADLOCK as a potential culture-free diagnostic for urinary tract infections (UTIs), achieving 98.0% reaction-level accuracy. At the assay level, 95.3% (41 of 43) were partially or perfectly concordant with gold-standard detection of both species and resistance genes. To our knowledge, this represents the first demonstration of the CRISPR-Cas13a diagnostic platform on clinical bloodstream infections to date and supports BADLOCK’s potential as a practical and scalable solution for rapid pathogen and resistance gene detection in resource-constrained settings.

## INTRODUCTION

Antimicrobial-resistant (AMR) organisms are considered by the World Health Organization (WHO) to be one of the 10 greatest health care threats facing humanity (*1*). The burden of bacterial infections and AMR is most severe in low- and middle-income countries (LMICs), where the medical infrastructure necessary for their effective diagnosis and treatment is lacking (*2*–*5*). In 2019 alone, there were an estimated 1.27 million global deaths due to resistant bacterial infections (*2*), and this is projected to reach more than 8 million associated deaths annually by 2050 (*6*), resulting in increasing calls to improve diagnostic strategies for containing the epidemic (*7*–*9*). One of the most concerning clinical manifestations of AMR is in bloodstream infections (BSIs), which are among the most severe and life-threatening infectious syndromes (*10*, *11*). BSIs can be caused by a wide range of bacterial pathogens, but the mortality rate is significantly higher when resistant bacteria are involved (*10*, *12*, *13*), and the early identification of these pathogens can improve patient survival (*14*–*16*). Therefore, a low-cost method to rapidly detect bacterial species and their AMR genes in clinical samples would be an invaluable tool for clinicians.

The standard diagnostic workflow for BSIs involves several sequential steps, each requiring substantial time, specialized infrastructure, and trained personnel (Fig. 1A). In brief, blood from a patient with suspected bacteremia is inoculated into liquid culture media and incubated until microbial growth is detected. Positive blood cultures are then subcultured onto solid media, where individual bacterial colonies are isolated for species identification. In well-resourced laboratories, this identification is typically performed using advanced mass spectrometry–based techniques, such as MALDI-TOF (matrix-assisted laser desorption/ionization–time of flight), while in lower-resource settings, traditional biochemical testing remains the primary method. Following species identification, the antimicrobial susceptibility testing (AST) is conducted either with automated systems that assess growth inhibition in the presence of antibiotics or with manual methods such as broth microdilution, Kirby-Bauer disc diffusion, or E-testing (*17*). Depending on bacterial growth rate, the availability of resources, and laboratory workflow efficiency, this entire process can take anywhere from 2 to 7 days (*18*), potentially delaying the initiation of targeted antimicrobial therapy.

**Fig. 1.**
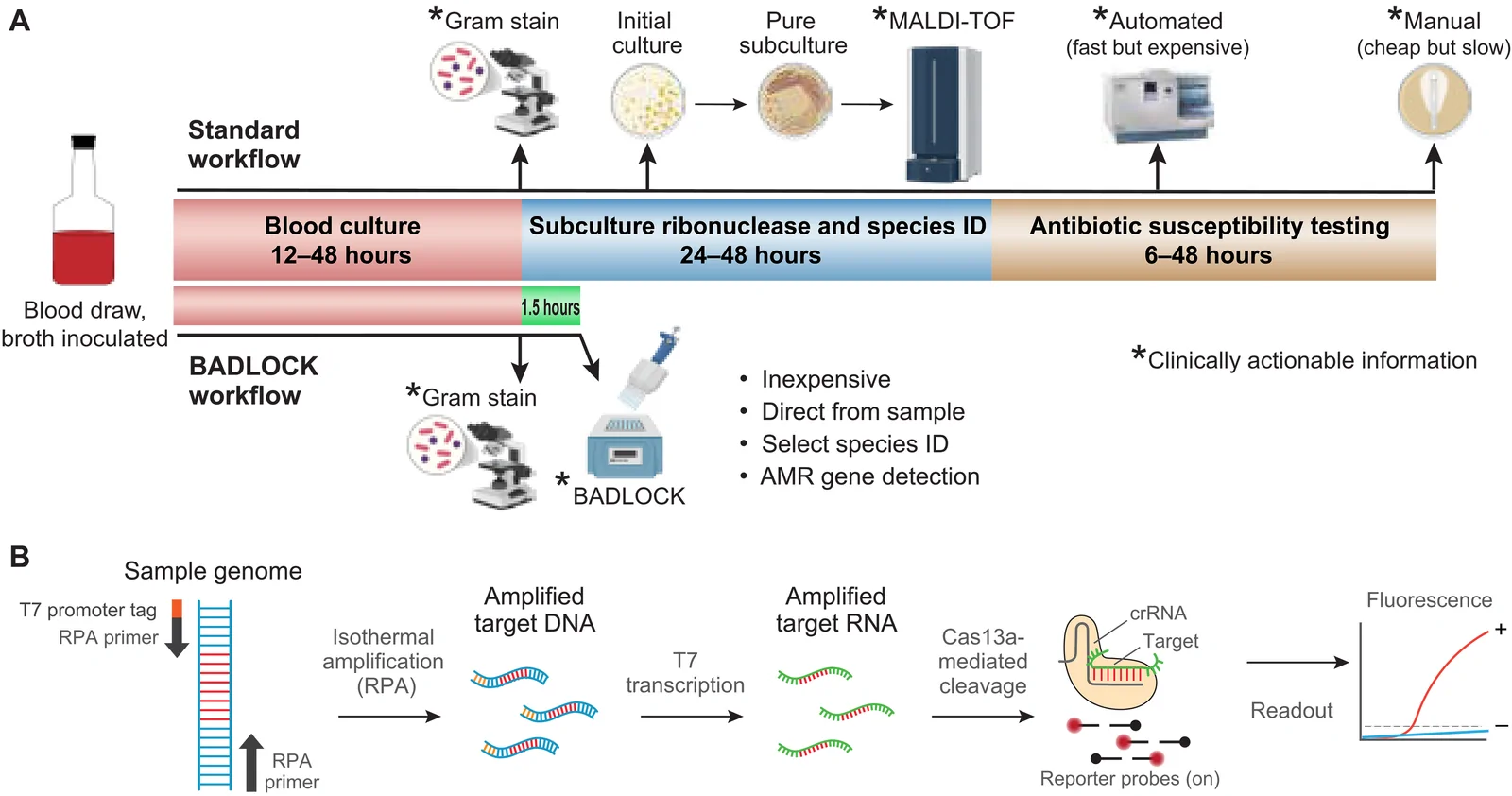
Blood culture workflow and SHERLOCK schematic. (**A**) Schematic representation of a standard clinical microbiology workflow for positive blood cultures in a well-resourced laboratory (top), demonstrating multiple sequential steps, extensive hands-on time, and reliance on complex instrumentation. In contrast, the BADLOCK workflow (bottom) demonstrates significantly reduced resource and instrument utilization and provides a much faster turnaround time for clinically actionable information. (**B**) Molecular mechanism of the SHERLOCK platform. Prespecified gene targets are amplified isothermally using RPA. The forward primer includes a universal T7 tag, enabling transcription of the amplified gene target into RNA. The CRISPR/Cas13a complex then binds to its cognate RNA target, triggering activation of collateral ribonuclease activity. Activated Cas13a subsequently cleaves synthetic RNA reporter molecules labeled with a fluorophore and quencher, separating the fluorophore from the quencher and generating a detectable fluorescent signal. Created in part on BioRender. R. Bhattacharyya (2026; https://BioRender.com/jf3f2e8).

In recent years, a growing number of alternative approaches have emerged to address the delays inherent in traditional workflows, including rapid, gene-based diagnostic platforms capable of simultaneously detecting bacterial species and AMR genes directly from cultures (*19*, *20*). These methods have demonstrated notable improvements in time-to-appropriate antibiotic therapy and patient survival (*16*). However, they can come with substantial challenges to widespread implementation, including high costs, a large infrastructural footprint, and the need for sophisticated laboratory facilities and specialized technical training. As a result, these advanced diagnostics are often inaccessible in underresourced regions, which disproportionately bear the global burden of bacterial infections, AMR, and associated mortality (*2*). This underscores the urgent need to develop rapid, low-cost bacterial diagnostic tools specifically designed for deployment in resource-limited settings (*6*, *17*, *21*).

Emerging nucleic acid detection technologies, such as SHERLOCK (specific high-sensitivity enzymatic reporter unlocking), offer promising solutions for addressing this challenge (*22*, *23*). SHERLOCK is a low-cost, portable, and highly programmable CRISPR-Cas13a–based system that enables the sensitive detection of nucleic acid sequences, including those from human pathogens (*24*–*26*). Its minimal laboratory infrastructure requirements make it particularly well-suited for deployment in low-resource environments (*24*). Leveraging the CRISPR-Cas13a enzymatic platform (Fig. 1B), we have developed a diagnostic assay, BADLOCK (bacterial and AMR detection by SHERLOCK), which enables the rapid identification of diverse bacterial species and key AMR genes directly from blood cultures. This approach streamlines the standard clinical microbiology workflow, reducing both the time and complexity required for pathogen identification while simultaneously providing critical information on clinically relevant gene content to guide targeted therapy. Designed in alignment with WHO recommendations for diagnostics in low-resource settings (*21*), BADLOCK is fast, is low cost, and requires minimal infrastructure, offering a practical and impactful solution for use in LMICs. In this proof-of-concept study, we demonstrate the capability of BADLOCK to accurately identify nine different Gram-negative rod (GNR) bacterial species directly from clinical blood cultures, which together account for more than 3.3 million global deaths annually (*3*) and represent more than 80% of GNR BSIs within our institution. We additionally demonstrate its ability to detect major epidemic resistance genes, underscoring its potential to address critical diagnostic gaps in global health. Last, we demonstrate that its flexible and robust format makes it a promising diagnostic tool for other biospecimens beyond blood cultures.

## RESULTS

### Assay target development

BSIs can be caused by a wide variety of highly diverse bacterial species (*11*), posing major challenges for the development of comprehensive gene-based diagnostics. To enhance our assay’s practicality and clinical utility, we focused on species that collectively account for ∼80% of Gram-negative BSIs within our local institution (fig. S1), which included *Escherichia coli*, *Klebsiella pneumoniae*, *Enterobacter cloacae* complex, *Citrobacter freundii*, *Serratia marcescens*, *Proteus mirabilis*, *Pseudomonas aeruginosa*, and *Acinetobacter baumannii*. We also included *Stenotrophomonas maltophilia* due to its intrinsic resistance to many commonly used antibiotics and treatment requirements that would affect clinical management (*27*). For the resistance gene panel, we targeted widespread β-lactamase genes that are strongly associated with resistance to broad-spectrum beta-lactams and have well-defined treatment recommendations in the 2024 Infectious Disease Society of America’s guidelines (*28*). These genes are linked to strains that are difficult to treat (*27*), resistant to many empiric regimens, and exhibit higher mortality (*13*, *29*). Specifically, we targeted the common extended-spectrum beta-lactamase gene *bla*_CTX-M-15_ and the carbapenemase genes *bla*_KPC_, *bla*_NDM_, and *bla*_OXA-48_. We elected to forego *bla*_VIM_ and *bla*_IMP_ due to their lower prevalence both globally (*30*) and within our study population (*31*, *32*). We employed three distinct strategies for target development: (i) targeting individual pathogenic species; (ii) detecting clusters of closely related species with similar clinical significance, such as the *E. cloacae* complex, thereby minimizing the overall panel size; and (iii) identifying conserved regions within clinically relevant antimicrobial resistance gene families (*26*) (Fig. 2, A and B).

**Fig. 2.**
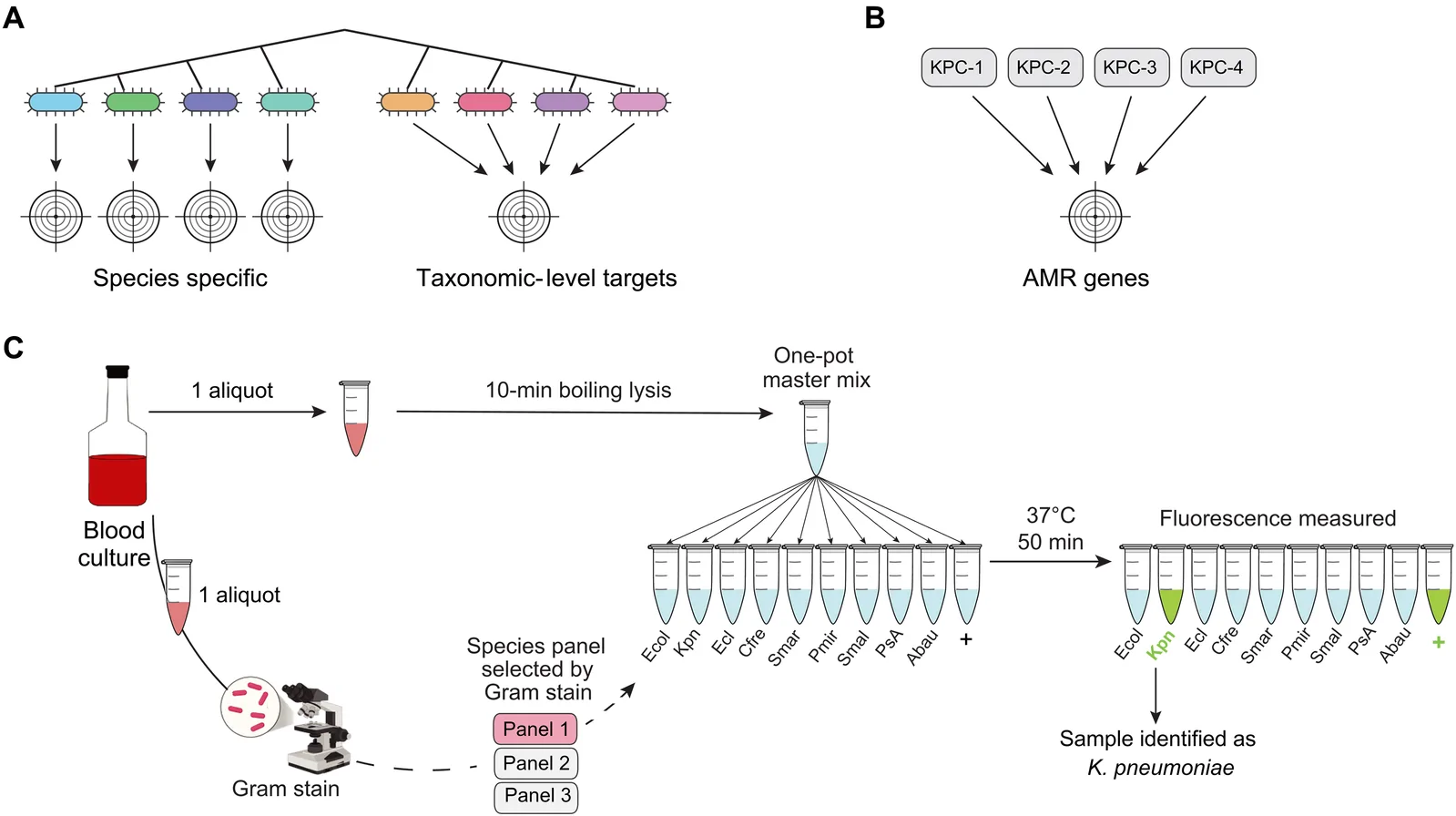
Targeting strategy and BADLOCK workflow. (**A**) Overview of the approach to species primer and guide development, showing that certain targets map to individual species, whereas others capture a group of closely related bacteria with similar clinical management pathways. (**B**) AMR gene targets are designed to capture most sequence variants for a given gene by targeting conserved regions. (**C**) BADLOCK workflow. Once blood cultures turn positive, a heat lysis step is performed, while a Gram stain is concurrently conducted. Gene panel selection is guided by Gram stain morphology, with this study focusing exclusively on GNRs. A single master mix is prepared and divided among different RPA primer and CRISPR guide pairs, each specifying a target. The reactions are incubated for 50 min, after which fluorescence values are measured to determine target detection. For simplicity, only Gram-negative species targets are shown here. Created in part on BioRender. R. Bhattacharyya (2026; https://BioRender.com/vz038yr).

The target design process has two layers of specificity, combining an isothermal amplification of a target region using recombinase polymerase amplification (RPA) with a 28–base pair (bp) CRISPR-Cas13a target within the amplicon (Fig. 1B). Our initial approach focused on the topoisomerase I gene for species-specific targets, as it was identified through a genome-wide scan to be highly conserved within species yet sufficiently divergent between them to enable precise detection with CRISPR guides (*26*). Using this strategy, we developed targets for four species but were unable to successfully amplify them with RPA in three others. In those, we identified genes from the literature that are conserved within and unique to the species of interest (table S1) (*33*–*35*). In *Escherichia coli*, we encountered a challenge, since RPA reagents are produced in the laboratory strain *E. coli* K12, leading to false positives. As a solution, we targeted the *chuA* gene, which is present in 80 to 90% of pathogenic *E. coli* strains but is absent from *E. coli* K12, a strategy used by other molecular diagnostic approaches using RPA to detect *E. coli* (*36*, *37*). To detect the six species that constitute the taxonomic grouping called the *E. cloacae* complex (*38*), we adapted a computational algorithm to identify conserved sequences shared among these species but absent from nontarget species (Methods). For AMR gene detection, we used previously identified conserved regions within the genes of interest (*26*). After identifying putative targets, we used ADAPT (*39*), a computational tool designed for CRISPR guide selection, and subsequently designed flanking RPA primers designed to amplify DNA segments containing the CRISPR target sites.

Each primer and guide combination was first tested on genomic DNA from at least two banked isolates per species of interest to verify functionality and specificity. This process was iterative, with primer and guide combinations refined as needed to ensure specificity and reproducibility. Using this approach, we successfully developed RPA primers and CRISPR guide targets for all species of interest (table S1). To identify targets within the select AMR genes, we designed RPA primers around known CRISPR guides (*26*) but found that the specificity for *bla*_NDM_ was low using the previously identified guide, requiring a guide redesign using ADAPT.

### One-pot assay design and optimization for use on blood cultures

To optimize the assay for clinical microbiology laboratory workflows, where minimizing hands-on technician time is critical, we developed a flexible one-pot approach that integrates RPA (*40*) with Cas13a-based detection in a single reaction. While similar approaches have used freeze-dried RPA pellets followed by master mix preparation (*25*), we found this method to be less flexible to varying sample input numbers. Instead, we adopted a liquid-based RPA format as our starting point, but initial experiments revealed that the standard liquid-based RPA reaction yielded less robust amplification when combined with the Cas13a detection buffers. To address this, we systematically varied buffer formulations, testing different relative concentrations and salt contents to identify an optimal mix. The best-performing formulation that maintained the final reaction volume, used magnesium acetate from the RPA reaction as the sole magnesium source, incorporated desalted reaction buffers, and used an RPA buffer at 0.66× the manufacturer’s recommended concentration (see Methods).

We next sought to transition from genomic DNA to bacterial cultures in laboratory media as the starting template for our assay. While detergent-based lysis methods have previously been used (*41*), we opted for a simple heat lysis step to simplify the workflow. The lower limit of detection (LOD) for broth cultures was determined to be 10^6^ colony-forming units (CFU) per milliliter for three representative species (fig. S2). The intended target biospecimen for our assay is positive blood cultures, which typically contain bacterial concentrations between 10^6^ and 10^8^ CFU/ml (*19*). Although bacterial cultures from LB media could be directly loaded into a reaction with reliable results, initial attempts to use undiluted blood culture specimens yielded low signal, likely due to interference from blood components within the sample matrix (*40*). To address this issue, we performed serial dilutions and identified that a 1:10 dilution of the blood culture reliably produced positive results.

To establish robust thresholds for positivity for each species target in clinical specimens, we used a previously validated approach (*42*), defining the threshold as six times the standard deviation above the mean relative fluorescence units (RFUs) measured for each CRISPR target in the absence of its corresponding template. Thresholds were calculated using 24 off-target clinical samples for the species targets and 12 off-target banked samples for the AMR gene targets (fig. S3). To ensure assay viability, a positive control was created using a single-stranded synthetic DNA sequence not predicted by BLAST (*43*) to be in any bacteria (Fig. 2C and table S1). Each BADLOCK assay consists of a predefined panel of gene targets that are evaluated in physically separate, parallel reactions, with each target occupying an individual reaction well (Fig. 2C). Assay setup requires less than 10 min of hands-on operator time and consists of a single heat lysis step, followed by distribution of a common master mix of off-the-shelf reagents into target-specific reaction wells. All enzymes are compatible with lyophilization and so theoretically do not require cold chain storage (*25*, *44*). The only consumables used are standard laboratory plasticware, including pipette tips and reaction tubes or plates, with no proprietary cartridges or microfluidic devices required. Assay execution requires only a basic heat block for isothermal incubation and a simple fluorometer for readout, thereby obviating the need for thermal cyclers or complex proprietary instrumentation (Fig. 2C).

To maintain consistency in terminology, we define an “assay” as the complete panel of targets run simultaneously and a “reaction” as an individual target within that panel. We use “false positive” and “false negative” to describe miscalls at the reaction level. At the assay level, we define “perfect concordance” as cases where all reactions within the assay were called in agreement with the clinical laboratory results, “partial concordance” as cases where at least one true positive was detected alongside one or more miscalls, and “discordant” as cases in which no true positives were detected or, in the case of off-panel species, when at least one false positive was called. In alignment with Food and Drug Administration (FDA) guidance on reporting diagnostic results for novel assays (*45*) and consistent with recently published evaluations for other multiplex panels (*46*), we report performance using positive percent agreement (PPA) and negative percent agreement (NPA). These metrics, analogous to sensitivity and specificity, are defined as follows: PPA is the proportion of true positives identified by the assay (concordant positives divided by all positive culture results), and NPA is the proportion of true negatives identified (concordant negatives divided by all negative culture results). We used an intent-to-diagnose standard in reporting our results to ensure the most conservative estimate of performance (*47*).

### BADLOCK’s species identification performance on a clinical cohort

We next evaluated BADLOCK on a cohort of frozen aliquots from 194 consecutive positive blood cultures identified as GNRs, collected by the Massachusetts General Hospital (MGH) clinical microbiology laboratory (Fig. 3A). These samples had undergone the standard clinical microbiology workflow, including Gram-stain morphology, bacterial species identification, and antimicrobial resistance testing. Among the cohort, 157 specimens matched at least one species included in our assay panel, while 37 specimens contained exclusively off-target species (fig. S1). In addition, we specifically wanted to interrogate polymicrobial samples (that is, when more than one species are causing bacteremia and grow from blood culture) as this is a known challenge in clinical microbiology diagnostics (*48*). In total, 18 specimens were polymicrobial; 8 contained one on-panel species, 6 contained two, and 4 contained no on-panel species.

**Fig. 3.**
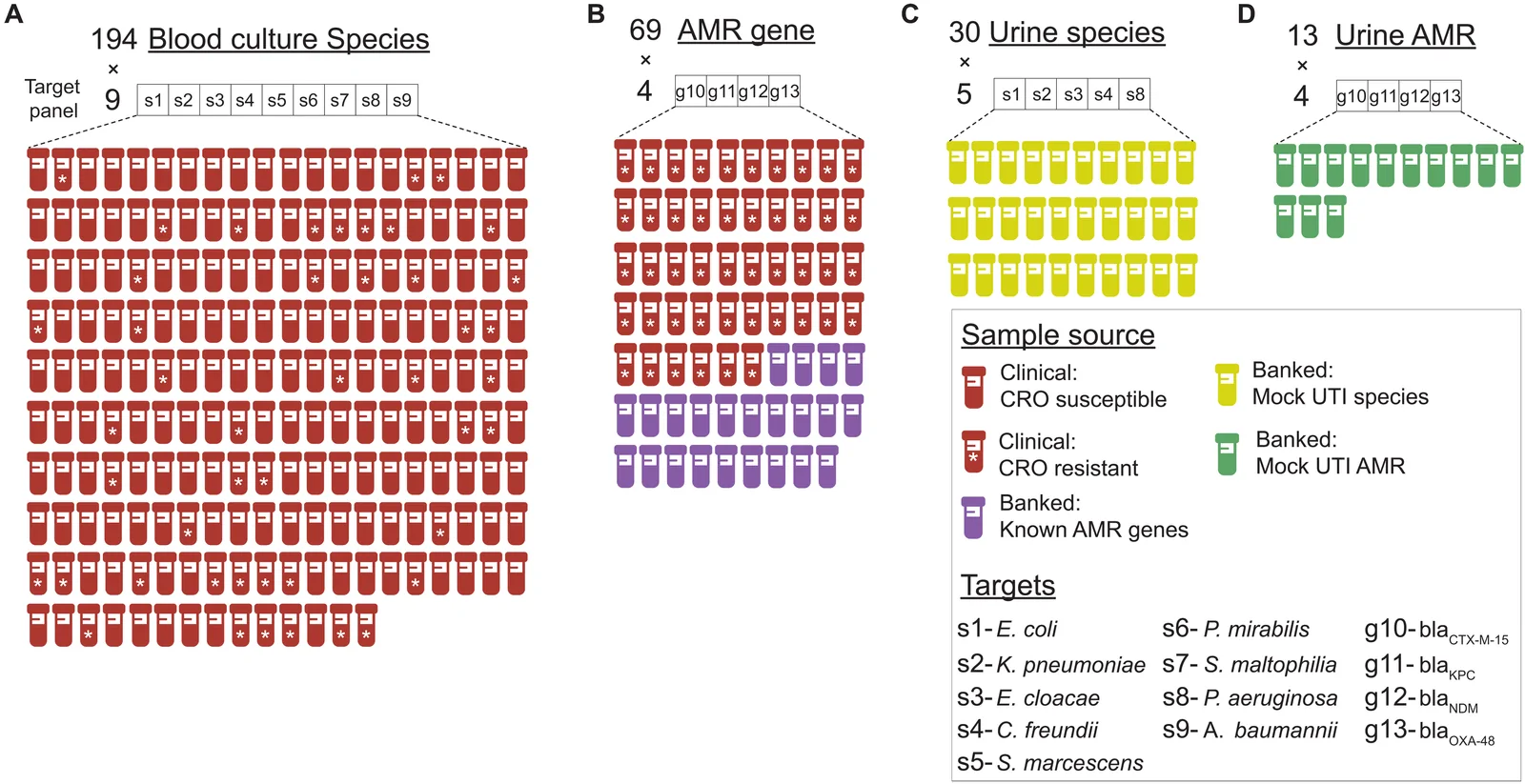
Cohort overview and associated CRISPR targets. Summary of each experimental group evaluated in this study, including (**A**) species testing on clinical blood culture specimens, (**B**) AMR gene testing on both clinical samples and banked laboratory isolates, (**C**) species testing on mock UTI samples created from banked isolates, and (**D**) AMR testing on mock UTI samples also created from banked isolates. Each cohort is annotated with the specific CRISPR targets used, illustrating the number and distribution of species- and resistance gene-level detection across study phases. Each sample is tested for all species targets within the target panel simultaneously, which are represented in the boxes above each cohort. “CRO” is the abbreviation of the third generation cephalosporin ceftriaxone.

Because each target is processed independently in separate reactions within the assay (Fig. 2C), these 194 samples amounted to 1746 individual reactions; reaction-level calls were correctly made in 97.7% (1705 of 1746) instances across the cohort (Fig. 4 and fig. S4). Among the 158 samples containing species targeted by BADLOCK, at least one target was correctly identified in 88.0% (139 of 158) of cases. Across the cohort on initial testing, there was perfect or partial concordance with subculture and MALDI-TOF in 88.1% (171 of 194) of cases, with 157 assays (80.9%) demonstrating perfect concordance and 14 (7.2%) with partial concordance. There were a total of 19 false positive reactions (across 17 samples) and 22 false negatives, with two samples containing both (fig. S4). BADLOCK identified at least one species in 100% (6 of 6) of polymicrobial cultures containing multiple on-panel species and both species in 50% (3 of 6) of cases. It identified 62.5% (5 of 8) in those polymicrobial samples containing a single on-panel species. In the 37 samples containing only off-panel species, BADLOCK detected no organisms in 34 (91.9%), indicating selectivity for intended targets (fig. S4). Individual performance characteristics for each RPA primer/CRISPR guide combination within the clinical cohort were calculated and varied across targets, with PPA ranging from 60% (*S. maltophilia*) to 100% (*P. aeruginosa* and *P. mirabilis*). The NPA, conversely, was high across the cohort, ranging from 97.7 to 100% across species targets (Fig. 4 and table S2).

**Fig. 4.**
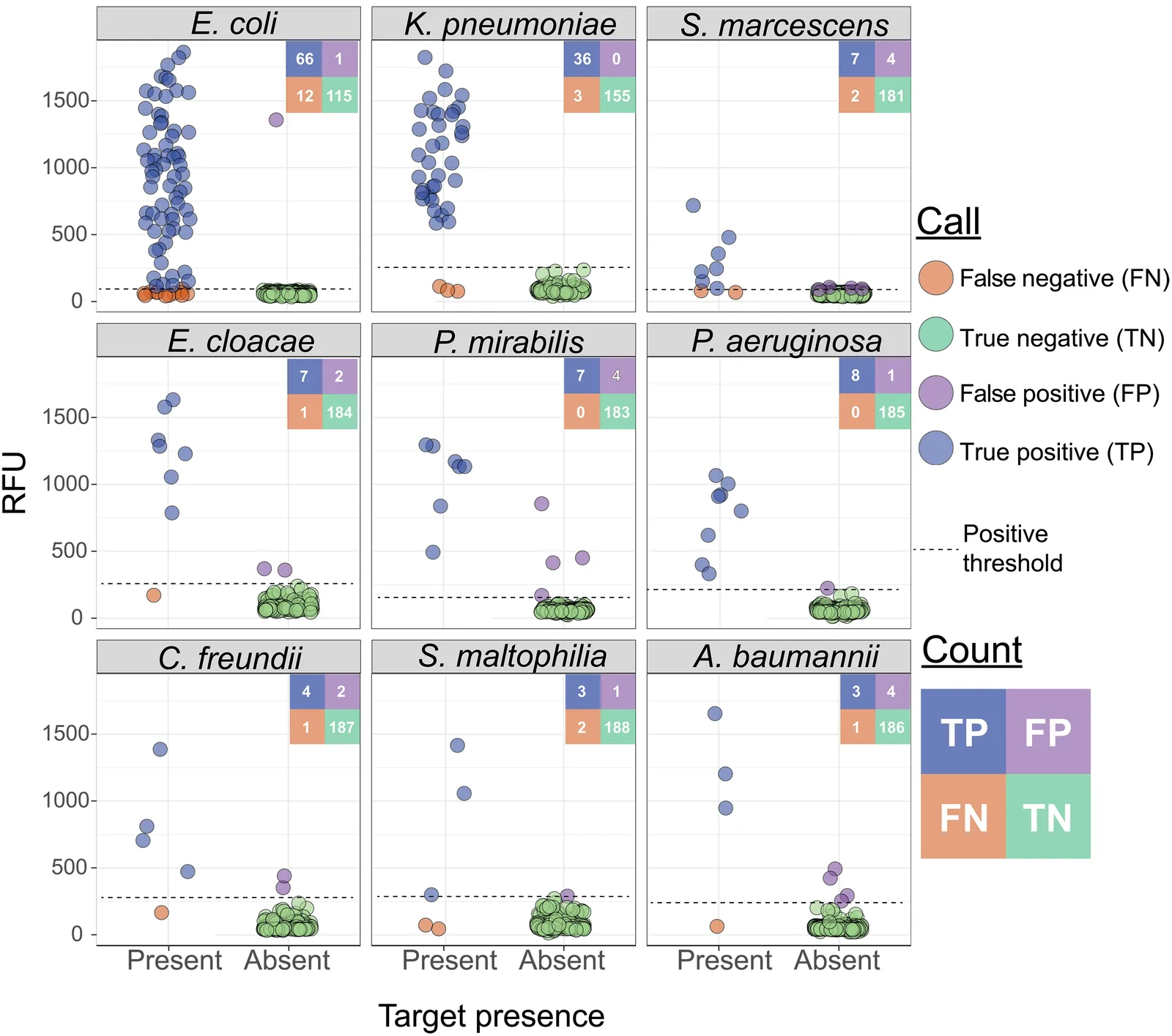
BADLOCK species panel performance on positive blood culture samples. Each panel displays RFU values for the corresponding species target across all 194 samples. A confusion matrix summarizing the total counts for each call is included for each species. Ground truth (*x* axis) was determined by routine testing in the clinical microbiology laboratory using the MALDI-TOF platform.

### Performance on antimicrobial resistance gene testing

To optimize reagent usage in this pilot study, we focused our testing on clinical samples known to be resistant to third-generation cephalosporins (which are more likely to harbor the ESBL or carbapenemase genes targeted by our assay), resulting in a final testing cohort of 46 clinical samples (Fig. 3B), only one of which was carbapenem-resistant. Polymerase chain reaction (PCR) was performed for all resistance gene targets in the ceftriaxone-resistant cohort to establish a ground truth for gene presence, and long-read whole-genome sequencing was conducted on a subset of nine samples for further validation. We achieved 96.2% accuracy (177 of 184) at the reaction level, and perfect concordance in 84.8% (39 of 46) of assays, with no partially concordant assays and 15.2% (7 of 46) discordance. We had 100% PPA for all AMR gene targets and 100% NPA for all carbapenemase genes but notably had 7 false-positive reactions for the *bla*_CTX-M-15_ gene target (Fig. 5 and fig. S5, left).

**Fig. 5.**
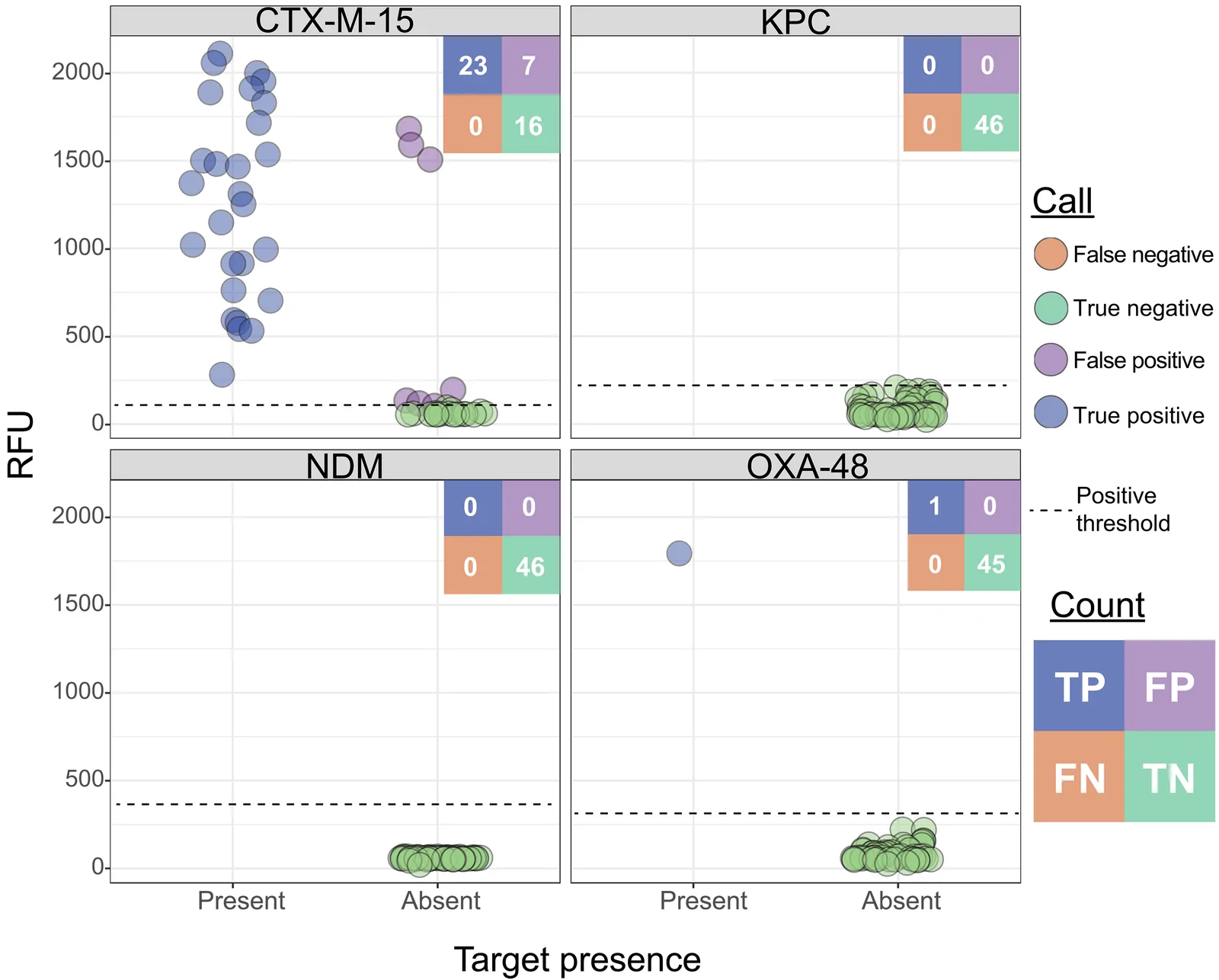
BADLOCK AMR gene testing performance on clinical samples. Each plot shows results for each individual target from the BADLOCK AMR gene testing panel run on the clinical cohort of positive blood culture specimens, limited to those 46 samples resistant to ceftriaxone, with gene content (*x* axis) validated by PCR testing or whole-genome sequencing.

Given the low prevalence of carbapenem resistance in the MGH clinical population (*15*), we supplemented our cohort with 23 banked carbapenem-resistant isolates with known beta-lactamase content (Fig. 3B and table S3), identified through prior sequencing efforts (*49*). Within this cohort of banked strains, BADLOCK had a correct reaction-level call in 98.9% (90 of 92) cases, resulting in perfect concordance in 91.3% (21 of 23) of assays and partial concordance in the remainder. The only two miscalls were from two false positives just above the threshold line (Fig. 6). Nine samples contained two on-panel genes, and BADLOCK correctly identified both genes in 100% (9 of 9) of cases (fig. S5, right).

**Fig. 6.**
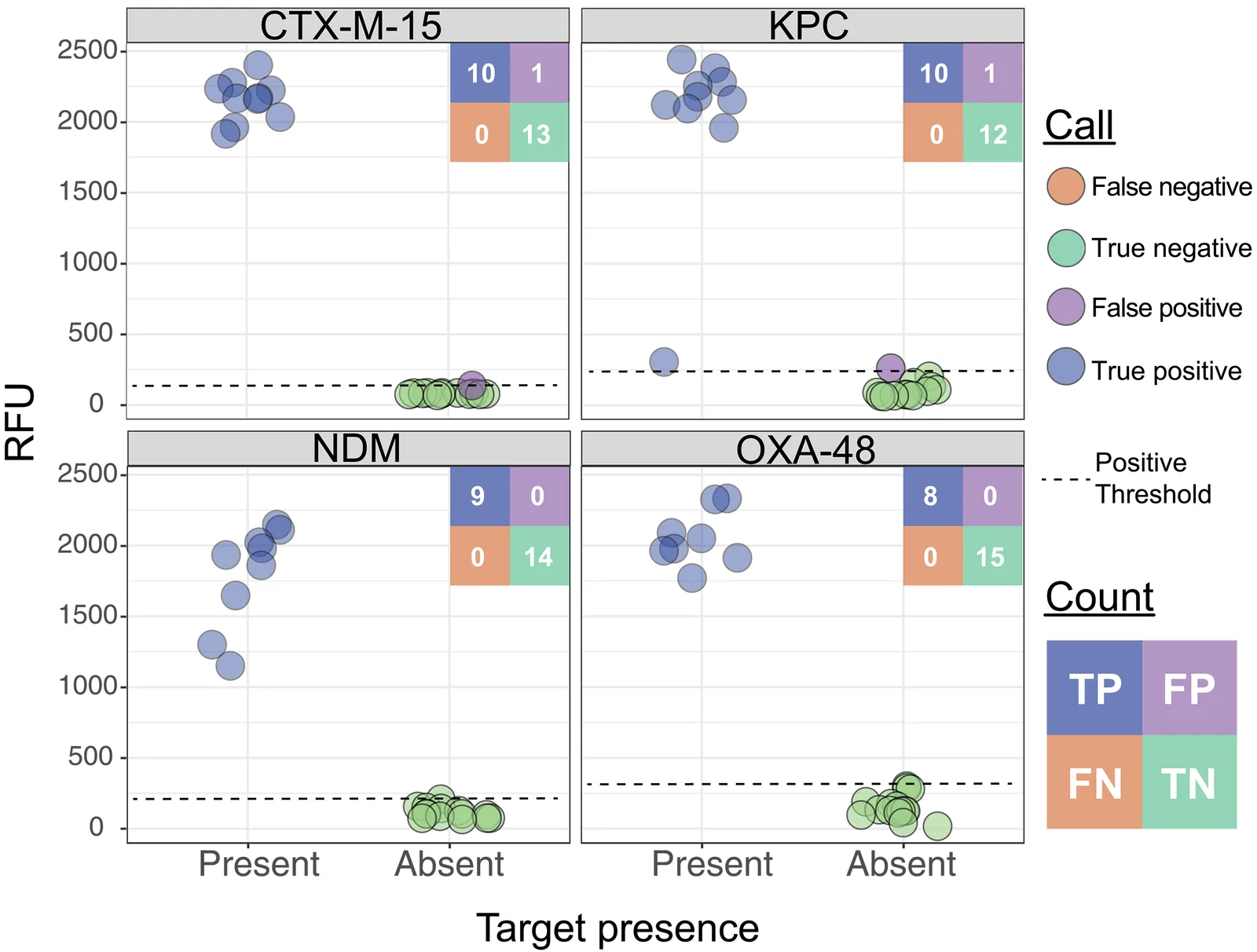
BADLOCK AMR gene testing performance on banked samples. Each plot shows results for each individual target from the BADLOCK AMR gene panel run on banked clinical laboratory strains with known genetic backgrounds (binned on *x* axis), validated by whole-genome sequencing.

### Investigation of discrepancies

We identified three distinct error modes contributing to discrepancies: (i) false negatives, where BADLOCK was negative but the gold-standard test was positive; (ii) false negatives due to absence of the molecular target, specifically the *chuA* gene in *E. coli;* and (iii) false positives, where BADLOCK was positive but the gold-standard test was negative. To investigate discordant results, repeat testing was performed as an internal quality control step (see Methods). Across species and AMR detection cohorts, 64% of discordant results were attributable to target absence (*chuA*-negative *E. coli* isolates, *n* = 11) or were resolved following a single repeat assay run (*n* = 21).

Of the 22 total false negatives, 12 were in *E. coli*, where we had to target a nonconserved gene (*chuA*) that is not present in the laboratory strain *E. coli* K12 used to make the RPA reagents. On the basis of reported likelihood of *chuA* carriage, we anticipated ∼15% of our *E. coli* samples would be falsely negative (*37*), which was consistent with our final detection rate of 84.6% (66 of 78). Of the 12 false-negative *E. coli* samples, PCR confirmed that the *chuA* gene was absent in 11. In the remaining case (sample 106), where *chuA* was present, repeat testing yielded a robust positive result (fig. S6). The remaining 45.5% (10 of 22) of false negatives were in non–*E. coli* species, and three of these resolved with a single repeat run (fig. S6). In two cases, the samples were polymicrobial, potentially indicating low-abundance species within the sample (where the blood culture may have signaled positive due to another species).

False positives were most frequently observed in *A. baumannii*, *S. marcescens*, and *P. mirabilis*, with each species accounting for four cases. We had previously used *A. baumannii* as a positive control during assay development, raising the possibility of amplicon contamination from prior runs as the source of these false-positive calls. This suspicion was supported by the fact that all *A. baumannii* false positives resolved when the assay was repeated at a later time point with fresh reagents (fig. S7). In all four *S. marcescens* false positives and in the *P. aeruginosa* and *S. maltophilia* miscalls, the RFU values were just above the threshold. All discrepancies but one resolved upon repeat testing, with the single persistent false positive for *S. marcescens* (sample 195) remaining positive but right at the threshold (see the “Threshold optimization” section below).

All seven discordant AMR results in the clinical sample cohort were false positives for *bla*_CTX-M-15_. Of these, three had very high RFU values, while four were only marginally above the positivity threshold (Fig. 5). One of the three high-RFU samples fully corrected on repeat testing, while two showed reduced signal but remained just above the threshold (fig. S8). Notably, all three of these samples had been batched and run together during the initial testing, raising suspicion of a cross-contamination event. For the AMR gene testing on banked isolates, the two discordant calls were a single false positive for *bla*_CTX-M-15_ and one for *bla*_KPC_, both of which occurred right at the threshold and corrected upon repeat testing (fig. S8).

### Threshold optimization

We set the initial positivity thresholds at 6 standard deviations above the mean of RFU values from off-target samples run prior to this study, but as more data was generated after running the entire sample set, we ran a post hoc receiver-operator curve (ROC) analysis to optimize thresholds within our cohort (fig. S9). ROC curves help identify optimal diagnostic thresholds by visualizing the trade-off between sensitivity and specificity across all possible cutoff values, enabling flexible threshold selection tailored to each diagnostic test (*50*). We evaluated the performance of each species- and gene-specific assay, comparing RFU values against known true-positive and true-negative classifications. Before analysis, we excluded false positive samples suspected to be caused by contamination in the initial reaction, defined as those with repeat RFU values more than threefold lower than the initial run (figs. S7 and S8), as well as *E. coli* samples lacking the *chuA* gene, as these would confound a ROC analysis (i.e., samples lacking the molecular target should not be used to set optimal thresholds for evaluating performance). Most targets demonstrated excellent diagnostic accuracy, with area under the curve (AUC) values ≥ 0.94 for all but one target. These assays showed near-perfect sensitivity and specificity at their respective optimal thresholds, as defined by Youden’s Index (*51*). In contrast, the *S. maltophilia* assay had lower overall discriminative ability (AUC = 0.72) due to limited sensitivity despite perfect specificity.

### Proof-of-concept test on mock urinary tract infections

Urinary tract infections (UTIs) have higher concentrations of bacteria than BSIs, potentially removing the need for a culture step and enabling direct-from-sample detection with BADLOCK. To evaluate this, we tested a subset of targets representing common urinary pathogens, including *E. coli*, *K. pneumoniae*, *E. cloacae*, *C. freundii*, and *P. aeruginosa*. Mock UTI samples were generated by culturing individual bacterial isolates and creating a dilution series for each species in filtered and pooled human urine (Methods). In our initial tests, BADLOCK successfully detected *E. coli*, *E. cloacae*, and *C. freundii* at concentrations below the established clinical threshold for infection, 10^5^ CFU/ml (fig. S10) (*52*, *53*), demonstrating promising sensitivity for direct-from-sample application. However, for *K. pneumoniae* and *P. aeruginosa*, the LOD was higher than the clinical threshold required for diagnosing infection. To improve sensitivity, we tested multiple approaches, including increasing template input volume, modifying reagent mixes, incorporating detergent-based lysis, and using centrifugal concentration. We found that using centrifugal concentration significantly enhanced sensitivity, enabling detection of all urine species targets at 10^5^ CFU/ml. Using the updated protocol, we re-established detection thresholds for urine samples as described above (fig. S11).

We next generated mock UTI samples, including at least five replicates for each gene target (including both species and AMR genes) and five samples containing off-panel species, each spiked at 10^5^ CFU/ml. This resulted in a total of 43 assay runs, comprising 30 species assays and 13 AMR gene assays (Fig. 3, C and D). At the reaction level, we achieved 98.0% concordance (198 of 202), whereas at the assay level, we achieved perfect concordance in 90.6% assays (39 of 43), partial concordance in 4.7% (2 of 43), and discordant results in 4.7% (2 of 43). The PPA was 100% for all targets except *K. pneumoniae* and *bla*_NDM_, which each had a PPA of 80% (corresponding to a single false negative each). The NPA was similarly high, with 100% for all targets except *E. cloacae* and *bla*_NDM-1_, which had 96.0 and 87.5% NPA, respectively (Fig. 7). Each of the four discrepancies was resolved with a single repeated run. No off-panel species yielded signal above the detection threshold, further supporting the assay’s specificity (fig. S12). This proof-of-principle shows that BADLOCK can be applied directly to a urine matrix at clinically relevant bacterial loads to reliably identify both bacterial species and AMR genes.

**Fig. 7.**
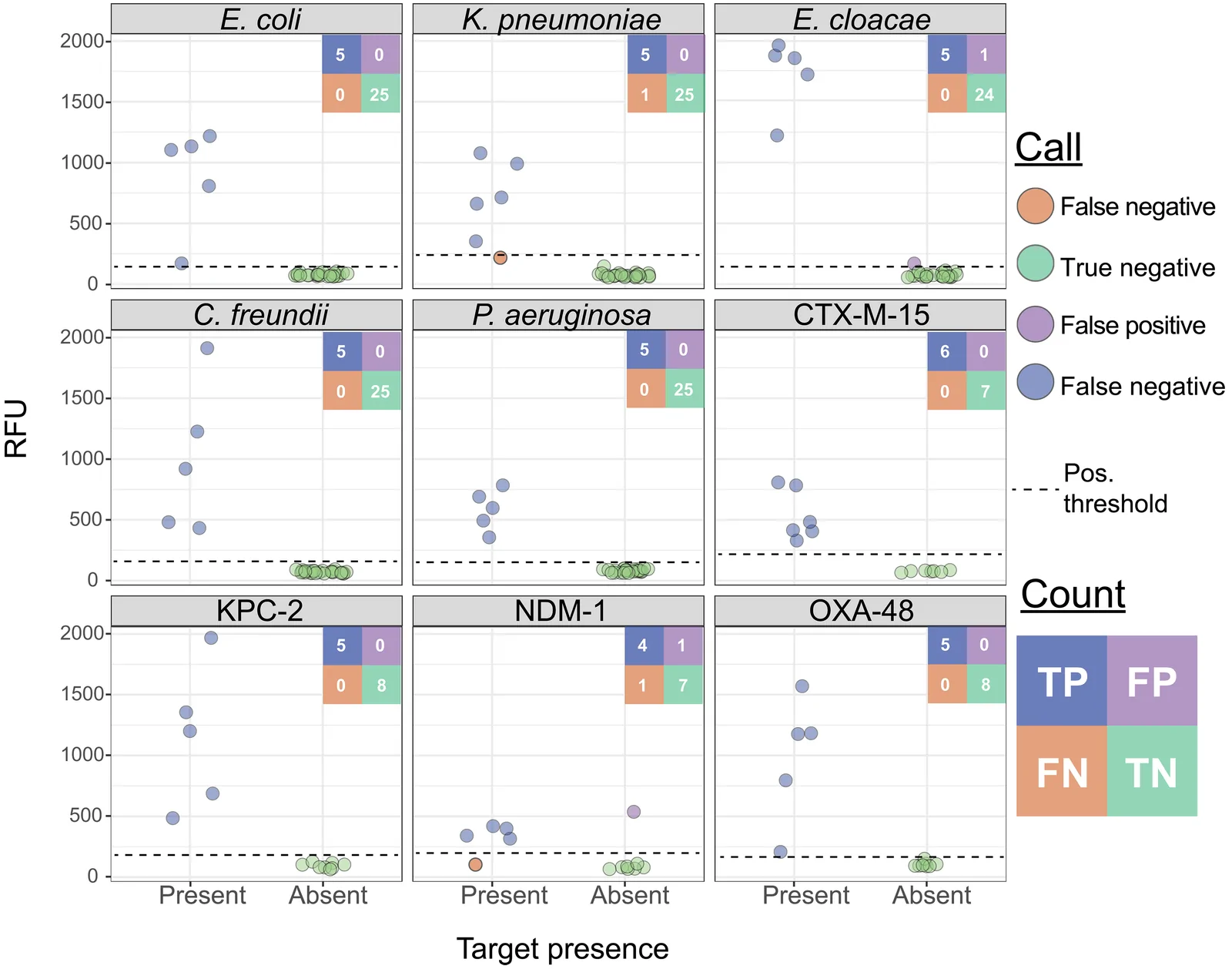
BADLOCK testing on mock urine samples. Each plot shows results for each individual target from BADLOCK testing of both species and AMR genes from mock urine samples spiked with bacteria of known species identity and resistance gene content.

## DISCUSSION

Here, we present BADLOCK, an inexpensive and adaptable diagnostic system designed to rapidly identify a panel of bacterial species that collectively account for more than 3 million global deaths annually (*3*), as well as major antimicrobial resistance determinants with significant clinical implications. We validated its utility in a retrospective study of 194 clinical blood cultures and conducted a proof-of-concept demonstration on mock UTI samples, corresponding to phase 2a and 1d studies, respectively, within a recently proposed framework for evaluating novel bacterial diagnostic platforms (*17*). To our knowledge, this is the first study to evaluate a SHERLOCK-based diagnostic platform on clinical BSIs, representing a critical step toward translating CRISPR diagnostics into real-world tools for the detection of clinically significant diseases. Across all cohorts and targets, we ran 2224 individual reactions with 97.7% accuracy, reflecting the high performance of individual RPA primer and CRISPR guide combinations. Our assay-level concordance was more variable, with perfect or partial concordance ranging from 88.1% for species detection in clinical blood culture specimens to >95% in the mock UTI cohort. This distinction between overall assay concordance and reaction-level accuracy is important in modular diagnostic platforms like BADLOCK, where multiple targets are evaluated independently within a single assay and variability across targets and sample matrices can affect composite results.

Our findings demonstrate the potential of BADLOCK to address key challenges in diagnostic capacity for BSIs, particularly in LMICs. In many LMICs, the substantial up-front investments (*54*) required for clinical microbiology laboratories—combined with the need for specialized and highly trained personnel—often limit their integration into clinical workflows. As a result, fewer than 1% of clinical laboratories have AMR testing capacity in some regions (*55*). This forces reliance on syndrome-driven therapies and broad-spectrum antibiotics, contributing to inappropriate antibiotic use and accelerating antimicrobial resistance in precisely the settings that experience the most resistance (*2*, *6*). BADLOCK was specifically designed with these limitations in mind, aligning with the REASSURED criteria established by the WHO to prioritize affordability, simplicity, and minimal infrastructure requirements (*21*), beyond the need for up-front blood culture. In addition, the organism and AMR gene targets included are all directly linked to guideline-based management protocols, ensuring clarity about the interpretation of results. By providing rapid, actionable results in a near-point-of-care format, BADLOCK offers a feasible solution for improving diagnostic capacity in resource-limited settings. Its ability to deliver results with minimal infrastructure may help bridge the critical diagnostic gap in LMICs, supporting more targeted therapy and reducing reliance on the use of empiric and increasingly ineffective antibiotics.

While the ideal diagnostic for BSIs would eliminate the need for a culture step entirely, the low bacterial load typically present in these infections poses a significant challenge that has proven exceedingly difficult to overcome (*56*, *57*). To date, the one high-cost commercial assay we are aware of that produces direct-from-blood pathogen detection has undergone significant corporate restructuring, due in part to the extreme technical challenges of this approach and lower diagnostic accuracy in real-world clinical trials (*58*). Beyond these technical hurdles, direct-from-blood approaches face a structural economic limitation: because fewer than 10% of blood cultures ultimately return positive, deploying these assays broadly would increase the effective cost per actionable diagnosis by more than an order of magnitude, even before accounting for the technical challenge of detecting as few as 1 to 10 bacterial cells in 10 ml of blood.

Commercial multiplex PCR assays such as the BIOFIRE Blood Culture Identification 2 (BCID2) Panel (bioMérieux) (*20*) and the VERIGENE Blood Culture Nucleic Acid Tests (Luminex) (*59*) target a similar panel of organisms and resistance genes as BADLOCK and use positive blood cultures as an entry point, underscoring both the clinical relevance of these targets and the utility of positive cultures as an assay input. However, the high per-run cost, significant infrastructure, and proprietary instrumentation requirements of these assays make them prohibitively expensive for many hospitals, even in well-resourced settings. For example, a recent systematic review only identified one middle-income country where these systems were in use (*20*), and a subsequent evaluation of the BCID2 system in a hospital in Zimbabwe—supported by multiple European grants—explicitly cited cost as a major barrier to broader adoption (*60*).

In parallel, recent technological advancements such as the Mini-Lab (*61*) and other innovations (*62*) show promise for lowering the barrier for blood culture capacity in LMICs, presenting natural synergies with a platform like BADLOCK. A recent modeling study estimated that, depending on clinical context, rapid diagnostics on positive blood cultures are cost-neutral in LMICs at prices up to ∼$109 per test under conservative assumptions (*63*). In its current prototype form, BADLOCK’s off-the-shelf reagent costs fall substantially below this threshold. This promising cost profile, along with compatibility with existing clinical microbiology workflows and minimal infrastructure requirements, position CRISPR-Cas–based assays such as BADLOCK to enable broader adoption of rapid molecular diagnostics in settings where conventional platforms remain impractical while remaining relevant in well-resourced laboratories. Moreover, the increasing availability of low-cost, multiplex fluorometers (*44*) further lowers implementation barriers and supports the feasibility of deploying BADLOCK in diverse laboratory settings.

This study had several limitations. First, due to the technical constraint of using RPA reagents produced in a laboratory strain of *E. coli*, we had to target a gene that was missing from that laboratory strain and thus not fully conserved within the species. Given that *E. coli* is the most common bacterial species implicated in Gram-negative BSIs, including in our cohort, this limitation led to an expected reduction in assay sensitivity. However, this could be addressed in future iterations through manufacturing processes that eliminate *E. coli* DNA contamination prior to commercialization or produce reagents in a nonpathogenic species. Second, a subset of underperforming primer and guide pairs contributed disproportionately to discordant results. Post hoc ROC analyses showed that thresholding can be improved with larger datasets, although further guide optimization, especially for *S. maltophilia*, will likely be required. Future work will focus on guide design strategies to boost performance for these species and incorporate the optimized thresholds in a priori analysis. Third, this pilot was conducted within a single institution, which may limit the generalizability of the findings to other institutions or geographic regions. However, our targets, primers, and guides were selected and designed to regions conserved across all sequenced isolates of each species or gene family (*26*), which should mitigate the impact of this limitation. Fourth, while this initial pilot focused on GNRs, Gram-positive bacteria also represent a substantial global burden of infection; we are currently developing assays targeting these pathogens, but these fall outside the scope of the present study. An additional limitation was probable cross-contamination leading to false positives in nine samples that retested negative, likely related to initial batched sample processing. Future iterations will address this through process improvements, including the use of pre-made reaction master mixes. Last, we used frozen clinical specimens and did not formally interrogate the effect of freeze-thaw cycles. However, our target validation and LOD studies were performed using unfrozen broth cultures which are likely comparable to the media used in blood culture broth.

In summary, we developed a CRISPR-Cas13a–based prototype assay, termed BADLOCK, as a promising step toward expanding rapid, low-cost diagnostic capacity in resource-limited settings. By validating the assay on a large cohort of clinical positive blood cultures, we demonstrated its potential for accurate pathogen detection and resistance characterization. In addition, we explored its utility as a direct-from-sample diagnostic in urine, highlighting its adaptability and broader application in diagnosing infections from diverse sources. These findings underscore BADLOCK’s feasibility as a scalable, accessible diagnostic tool, addressing a critical gap in infectious disease management where rapid and affordable testing is most needed.

## METHODS

### Clinical samples and ethics statement

Clinical blood culture aliquots were deidentified before use in the research laboratory. Study protocol was reviewed and approved by the Institutional Review Board (IRB) under Mass General Brigham IRB protocol 2015P002215, which permits research use of deidentified residual clinical specimens without additional informed consent.

### CRISPR guide design and RPA primer validation

CRISPR guides were selected by inputting the desired target genes into ADAPT, which generates a ranked list of putative guide sequences. Once a candidate guide was selected, RPA primers flanking the target region were designed. Multiple forward and reverse primers were synthesized, and all possible pairwise combinations were tested to identify viable primer sets. These were evaluated using target genomic DNA as the template, with water included as a no template control. RPA reactions were set up in 0.2-ml PCR strip tubes using the TwistAmp Liquid Basic Kit (TwistDx). Each 250 μl reaction mix contained 10 μl of diluted and lysed bacterial template, 165 μl of TwistAmp 2× buffer (to yield a final 0.8× concentration), 22 μl of TwistAmp 20× Core Reaction Mix (1× final concentration), 44 μl of TwistAmp 10× E-Mix (1× final concentration), and 44 μl of a 4× deoxynucleoside triphosphate (dNTP) mix (containing 2.5 μM each dNTP; Takara Biosciences) to achieve a final dNTP concentration of 1 μM. Reactions were mixed gently by pipetting and incubated at 37°C for 50 min. Amplification products were purified using QIAquick column purification (QIAGEN) and visualized using Invitrogen E-Gel General Purpose Agarose Gels (2%) (Thermo Fisher Scientific), precast with SYBR Safe DNA stain. A 15-μl aliquot from each RPA reaction was loaded directly into the wells of a 12-well E-Gel cassette alongside 7.5 μl of a 100-bp DNA ladder (New England Biolabs) to assess product size. Gels were run using the E-Gel Power Snap Electrophoresis System (Thermo Fisher Scientific, catalog no. G8100) under default conditions for 10 min. Following RPA primer validation, DNA guides were synthesized (Integrated DNA Technologies) with universal T7 transcription tails and subsequently transcribed into RNA, as described elsewhere (*26*). Each RPA primer and CRISPR guide pair was then tested in a one-pot reaction using target genomic DNA as the template and water as a no-template control (see the “BADLOCK assay” section below for reaction conditions). Primer-guide pairs that generated high on-target RFU values and minimal background signal in the water control were advanced for further validation. This process was repeated iteratively until viable primer-guide combinations were identified for all targets of interest.

### *E. cloacae* species complex guide design

To identify conserved sequences shared within different subspecies of the *E. cloacae* complex (ingroup) but absent from nontarget species (outgroup), we adapted an algorithm from Thakku *et al.* (*42*). Briefly, for each ingroup or outgroup species, we downloaded up to 100 genomes that satisfied our criteria: complete assembly, latest version, and not anomalous. We then listed all the overlapping 100 mers (e.g., 1 to 100, 2 to 101, etc.) of the reference genome of *E. cloacae* and aligned each 100 mers to all ingroup and outgroup genomes using bowtie2 (*64*) with score-min = *L*,0,0 (i.e., exact match) for ingroup and score-min = *L*,0,−0.6 (i.e. up to ∼10 mismatches) for outgroup. One hundred mers that aligned, using these parameters, to all ingroup genomes and to none of the outgroup genomes were used downstream for guide design using ADAPT.

### Bacterial collection and standard clinical typing

Between October 2023 and April 2024, all sequentially collected blood cultures taken as part of routine clinical care at MGH that signaled positive on the BACTEC FX system (Becton Dickinson, Sparks, MD) and were GNRs by routine Gram staining were included. After staining, a 1-ml aliquot of the culture was stored at −80°C. The remainder of the sample then underwent routine clinical processing with solid-media subculturing and species identification via MALDI-TOF mass spectrometry (VITEK MS, version 3.2 in vitro diagnostic Knowledge Base, *bioMérieux*, Durham, NC) and AST performed on the VITEK 2 AST-GN81 Gram Negative Susceptibility Card (bioMérieux), with Clinical and Laboratory Standards Institute breakpoints from the M100-Ed31.

### BADLOCK assay

To limit reaction interference from blood products, 10 μl of sample was added to 90 μl of nuclease-free water. Heat lysis of the samples was performed by boiling at 95°C for 10 min. Each BADLOCK run consisted of 10 individual reactions that combine RPA amplification, T7 transcription, and Cas13a-mediated detection of the target. To do so, a single master mix was made combining the following reagents: 13 μl of the diluted and lysed bacterial template, 99 μl TwistAmp liquid basic 2× buffer (TwistDx) (0.8× final concentration), 13.2 μl of TwistAmp liquid basic 20× Core Reaction Mix (1× final concentration), 26.4 μl of TwistAmp liquid basic 10× E-mix (1× final concentration), 26.4 μl of a dNTP mix containing each dNTP at 2.5 μM (Takara Biosciences) for an aggregate dNTP concentration of 1 μM, 26.4 μl of salt-free cleavage buffer (40% by volume 1 M tris-HCl at pH 7.5, 10% by volume 100 mM dithiothreitol, and 50% by volume nuclease-free H_2_O), Cas13a resuspended in 26.4 μl of salt-free storage buffer (nuclease-free H_2_O with a final concentration of 50 mM tris-HCl at pH 7.5 and 5% glycerol) for a final Cas13a concentration of 45 nM, 10.56 μl of 25 mM rNTPs (New England Biolabs) for a final concentration of 1 mM, 7.9 μl of T7 polymerase (50,000 U/ml; New England Biolabs) for a final concentration of 1.5 U/μl, 6.6 μl of ribonuclease (RNase) inhibitor murine (40,000 U/ml; New England Biolabs) for a final concentration of 1 U/μl, and 4.125 μl of 8 μM RNase Alert Reporter V2 (Life Technologies). This master mix was vigorously mixed and 18.7 μl were split across each individual reaction well, which contained 0.5 μl of 20 μM RPA forward primer, 0.5 μl of 20 μM RPA reverse primer, and 1 μl of 450 nM CRISPR guide designed around the respective target of interest. Last, 1 μl of 280 nM magnesium acetate was added to reaction as the sole cofactor and salt added to the mix, for a final concentration of 12.4 nM. The assay was then run at 37°C for 50 min. When run on spectrophotometry, each sample was checked at a wavelength of 490 nm, and the RFUs were recorded.

### Pilot study

Clinical samples were processed in batches of up to four specimens per assay run, with each run defined as a single plate processed under identical reaction conditions. Operators (D.J.R., B.P.S., and S.B.) were blinded to species identification and antimicrobial resistance profiles during testing and primary interpretation. Each run included a positive control (table S1) to monitor assay performance. Aggregate assay outputs were reviewed periodically for quality control purposes without modification of predefined positivity thresholds. In accordance with an intention-to-diagnose framework, assays demonstrating discordant individual reactions were repeated once at the assay level for discrepancy investigation only, and initial results were retained for primary performance analyses.

### Creation of mock UTI samples and urine sample preparation

Stocks were streaked onto LB agar plates and incubated at 37°C for 24 hours. A single colony from each plate was inoculated into LB broth and cultured at 37°C with shaking until the optical density (OD) at 600 nm reached 0.250 to 0.500, as measured using a plate reader. Following growth, each bacterial culture was diluted in pooled, filtered human urine (Innovative Research) to a target concentration of 10^8^ CFU/ml, based on standard OD to CFU conversion values. This culture (10^8^ CFU/ml), prepared in a final volume of 100 μl, served as the starting point for a serial dilution series. All serial dilutions were carried out using the same pooled human urine as the diluent to maintain matrix consistency across all experimental conditions. Expected CFU values were confirmed through plating. For samples between 10^4^ and 10^6^ CFU/ml, 1000 μl was transferred into a 1.5-ml tube and centrifuged at 21,300*g* for 1.5 min to pellet the cells. Following centrifugation, 980 μl of the supernatant was carefully removed to achieve a 50× concentration of the bacterial sample. The pellet was then resuspended and heat-lysed at 95°C for 10 min, followed by cooling at 4°C for 2 min. This template was then carried forward in the same manner as described above.
