# Supplementary material for: A rapid, inexpensive diagnostic for bacterial pathogen and resistance detection in resource-limited settings
Source: Sci Adv. 2026 Oct 2;12(40):eaeb6630. doi: 10.1126/sciadv.aeb6630 (PMC13632719; doi:10.1126/sciadv.aeb6630)
Supplement: Supplementary file 1 [file sciadv.aeb6630_sm.pdf]

Science Advances
AAAS

Supplementary Materials for

A rapid, inexpensive diagnostic for bacterial pathogen and resistance detection in resource-limited settings

David J. Roach *et al.*

Corresponding author: David J. Roach, djroach@mgh.harvard.edu;
Roby P. Bhattacharyya, rbhatt@broadinstitute.org

Sci. Adv. **12**, eaeb6630 (2026)
DOI: 10.1126/sciadv.aeb6630

The PDF file includes:

Figs. S1 to S12
Tables S1 to S3
Legend for data file S1

Other Supplementary Material for this manuscript includes the following:

Data file S1

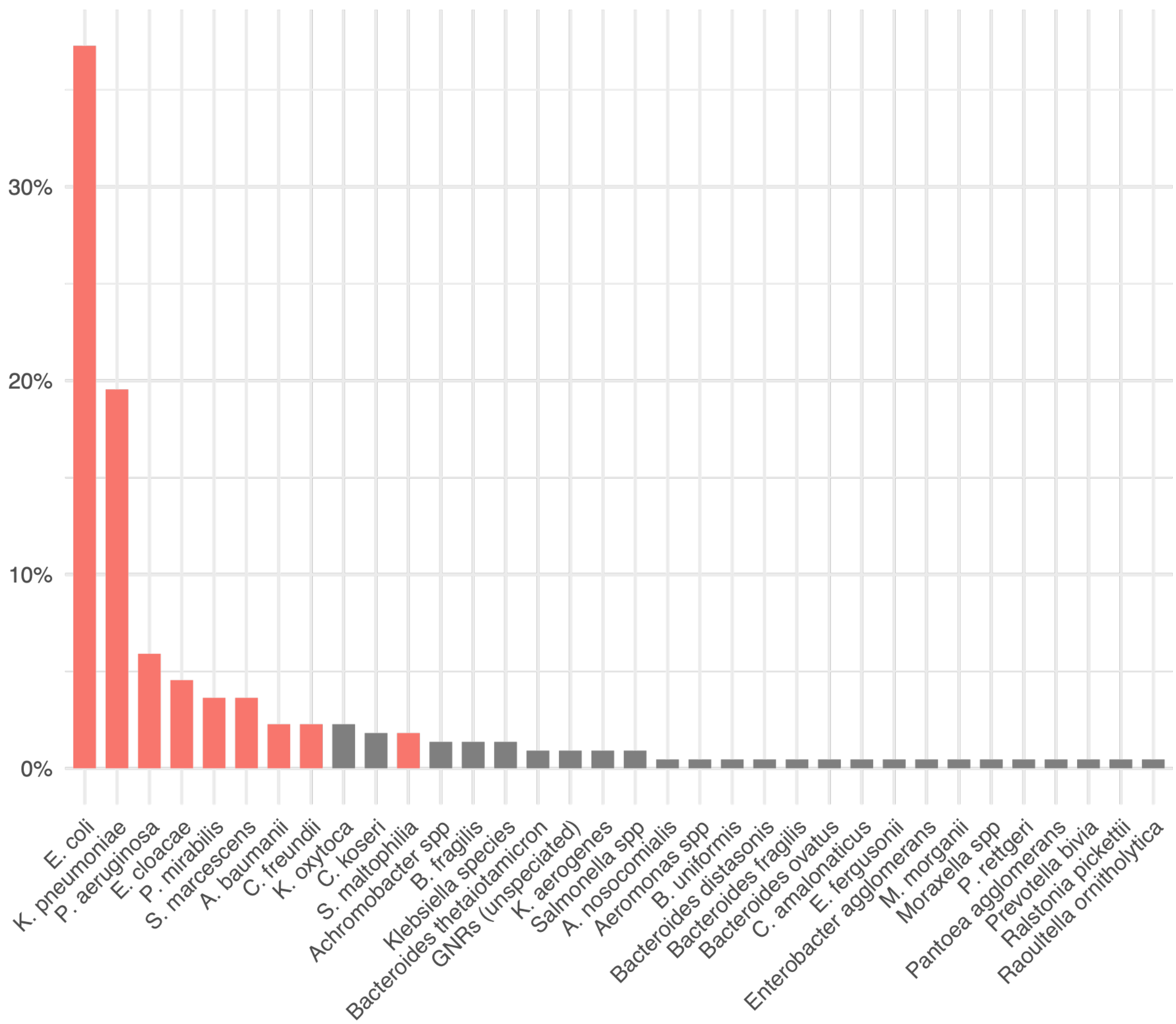

Figure S1. Proportional representation of gram-negative rod species from our entire clinical cohort. Species included in BADLOCK are shown in red.

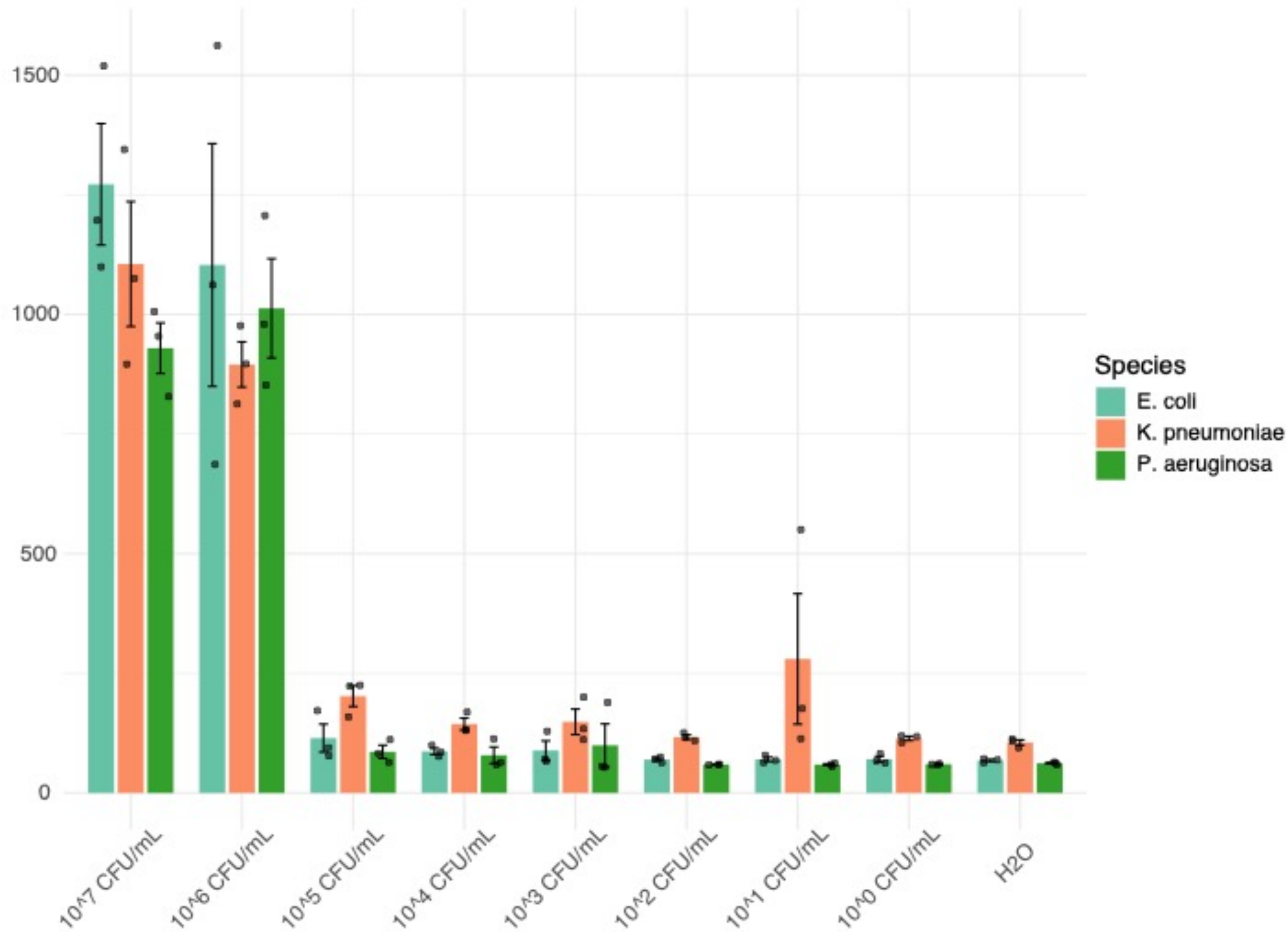

Figure S2. Limit of detection (LOD) analysis of the BADLOCK assay using LB-cultured isolates. RFU values (y-axis) are shown for three representative bacterial species across a serial dilution range measured in CFU/mL (x-axis). The BADLOCK assay reproducibly detects targets at concentrations of ~10^6 CFU/mL (corresponding to 10^3 CFU/uL), establishing this as the approximate lower limit of detection under these experimental conditions.

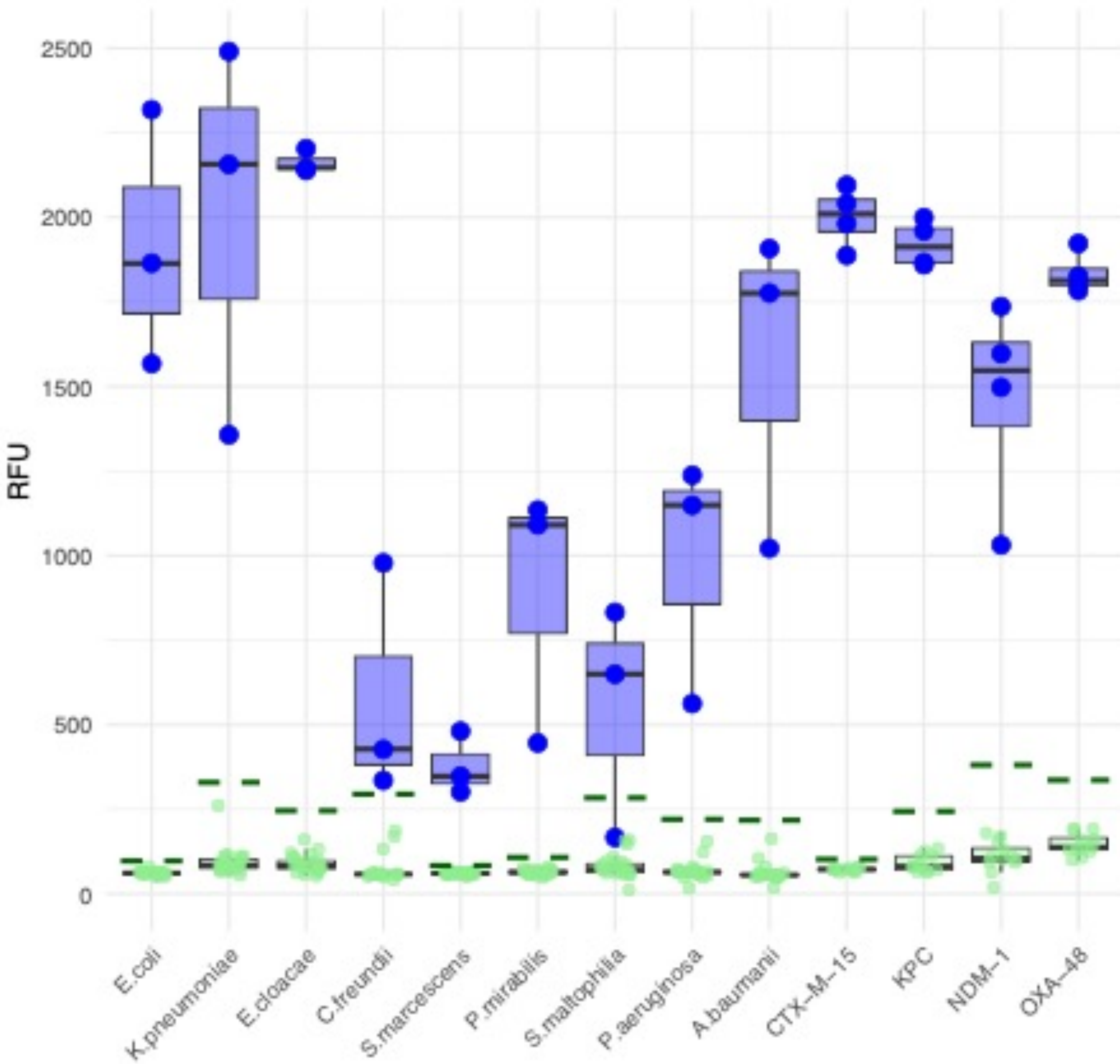

Figure S3. Threshold determination for species and antimicrobial resistance gene targets in the BADLOCK assay. Fluorescence values from technical replicates are shown for species targets using clinical samples, and for antimicrobial resistance (AMR) gene targets using banked clinical strains. The horizontal dashed line indicates the threshold for calling a positive result, defined as the mean plus six times the standard deviation of fluorescence values from 24 off-target samples for species reactions and 12 off-target samples for AMR gene reactions.

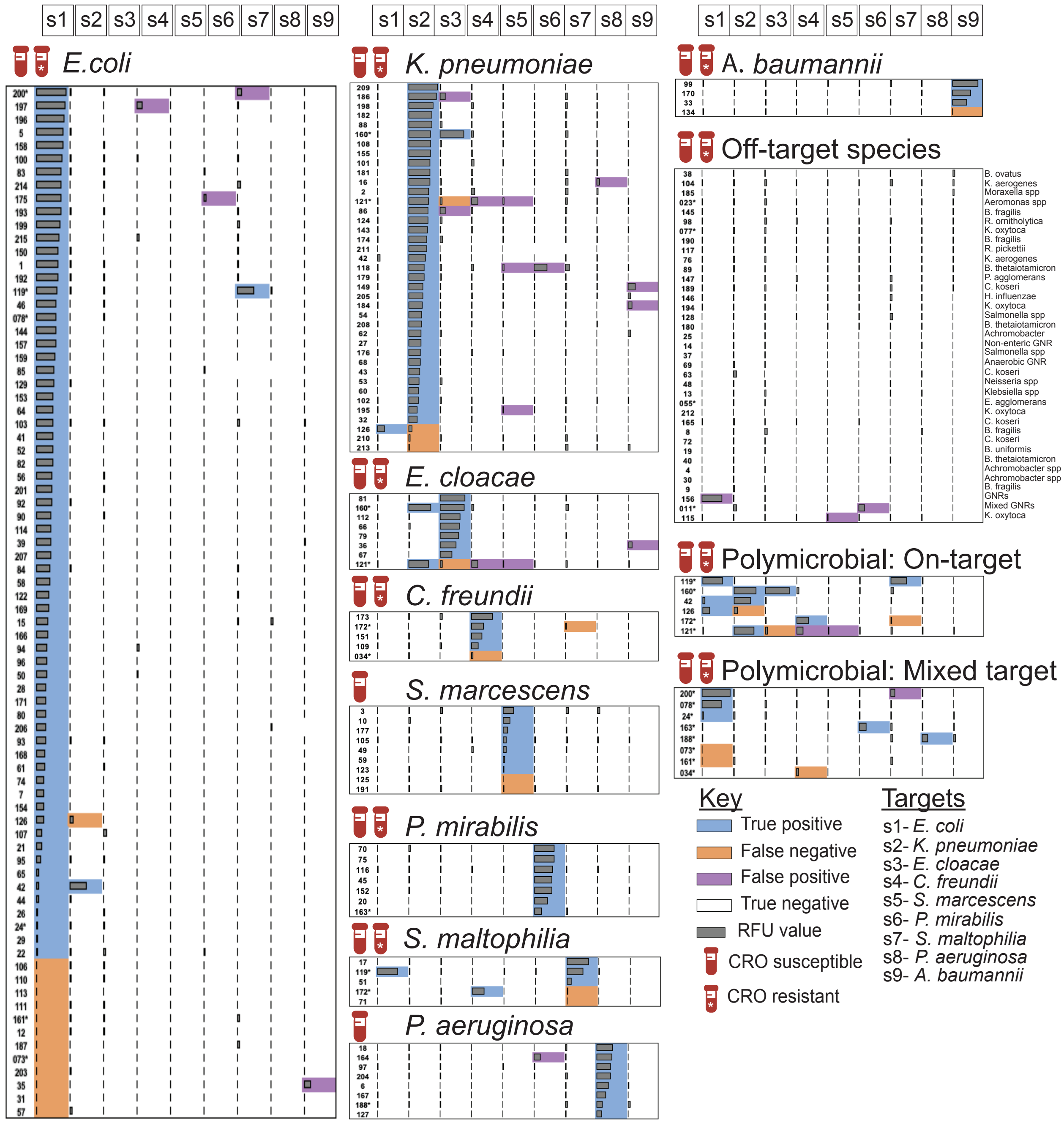

Figure S4. Consolidated visualization of BADLOCK species assay runs across all tested blood culture samples collected for this study. Each row represents an individual sample, and each column corresponds to a specific BADLOCK target with the assay layout shown in the boxes above and enumerated in the accompanying key. Samples are grouped by the species for which they were known positives as identified by the clinical microbiology laboratory's standard workflow, and within each group, ordered by RFU of the on-target reaction. Each cell in the grid reflects a single target reaction for a given sample, color-coded by accuracy (see Key). Overlaid grey bars represent the corresponding RFU values. Symbols adjacent to species names indicate the cohort origin of each sample and are harmonized with the legend in Figure 3. Only the first run for each reaction is shown here. Polymicrobial samples appear multiple times - once for each on-panel species present, and again in the "polymicrobial" section.

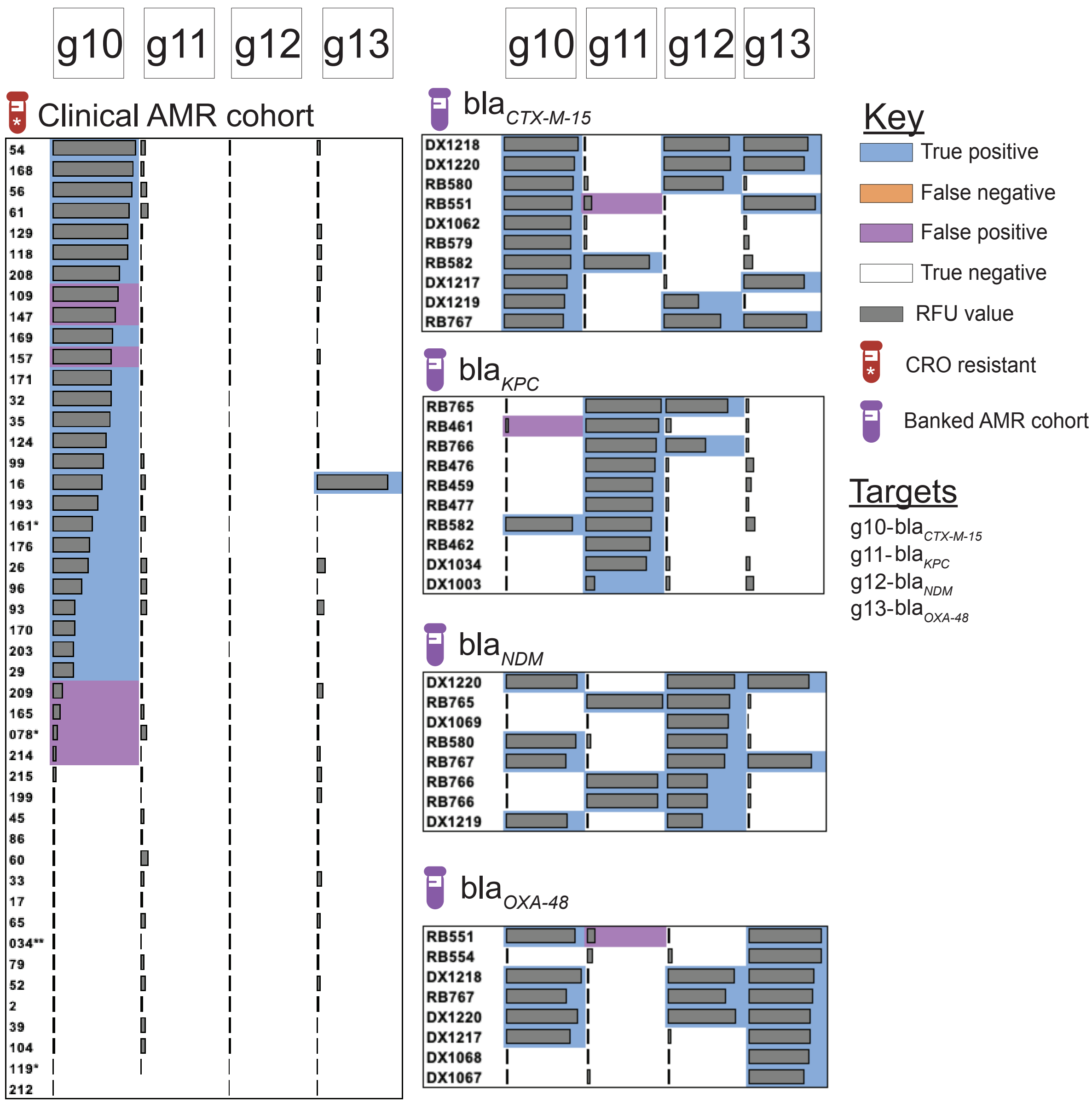

Figure S5. Consolidated visualization of BADLOCK AMR gene assay runs across all tested samples. Each row represents an individual sample, and each column corresponds to a specific BADLOCK AMR gene target, with the assay layout shown in the boxes above and enumerated in the accompanying key. Samples are grouped by the resistance gene known to be present by either PCR testing (clinical resistant cohort) or whole genome sequencing (banked cohort) (Table S3), and within each group, ordered by RFU of the on-target reaction. Each cell in the grid reflects a single target reaction for a given sample, color-coded by accuracy, with overlaid grey bars representing the corresponding RFU values. Symbols adjacent to gene names indicate the cohort origin of each sample and are harmonized with the legend in main-text Figure 3. Samples containing multiple AMR gene targets appear in each group for which a corresponding target was known to be present.

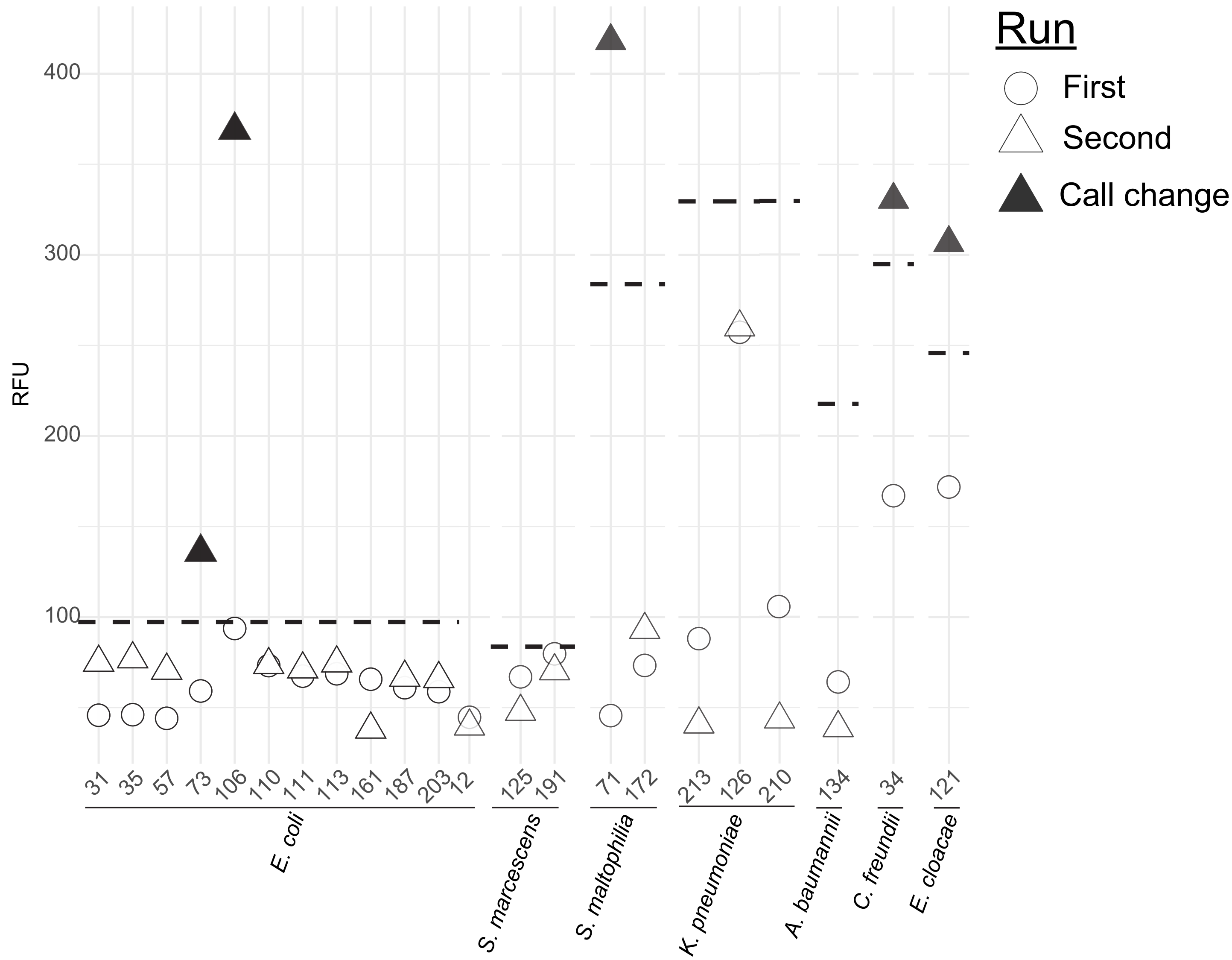

Figure S6. Repeat testing of falsely negative samples in the BADLOCK assay. Each point represents a clinical sample that was initially negative by BADLOCK but positive for the indicated species by the reference method. For simplicity, only the discordant targets are shown. Samples are displayed along the x-axis, grouped by species, with shape indicating whether the data correspond to the original or repeat run. The dashed horizontal line marks the species-specific RFU positivity threshold. A change in result classification occurs when a point crosses this threshold between runs, marked by a shaded triangle. 5 samples retested as concordant, while 17 remained discordant. Ten of these persistently false negatives were in *E. coli* while 7 were non-*E. coli* species, and 4 of these were from polymicrobial samples (IDs 172, 126, 34, and 121). One *E. coli* sample that retested as concordant was 106, which was confirmed to have the *chuA* gene target by PCR, while all other false negative *E. coli* lacked the target, including the other that retested positive (sample 73).

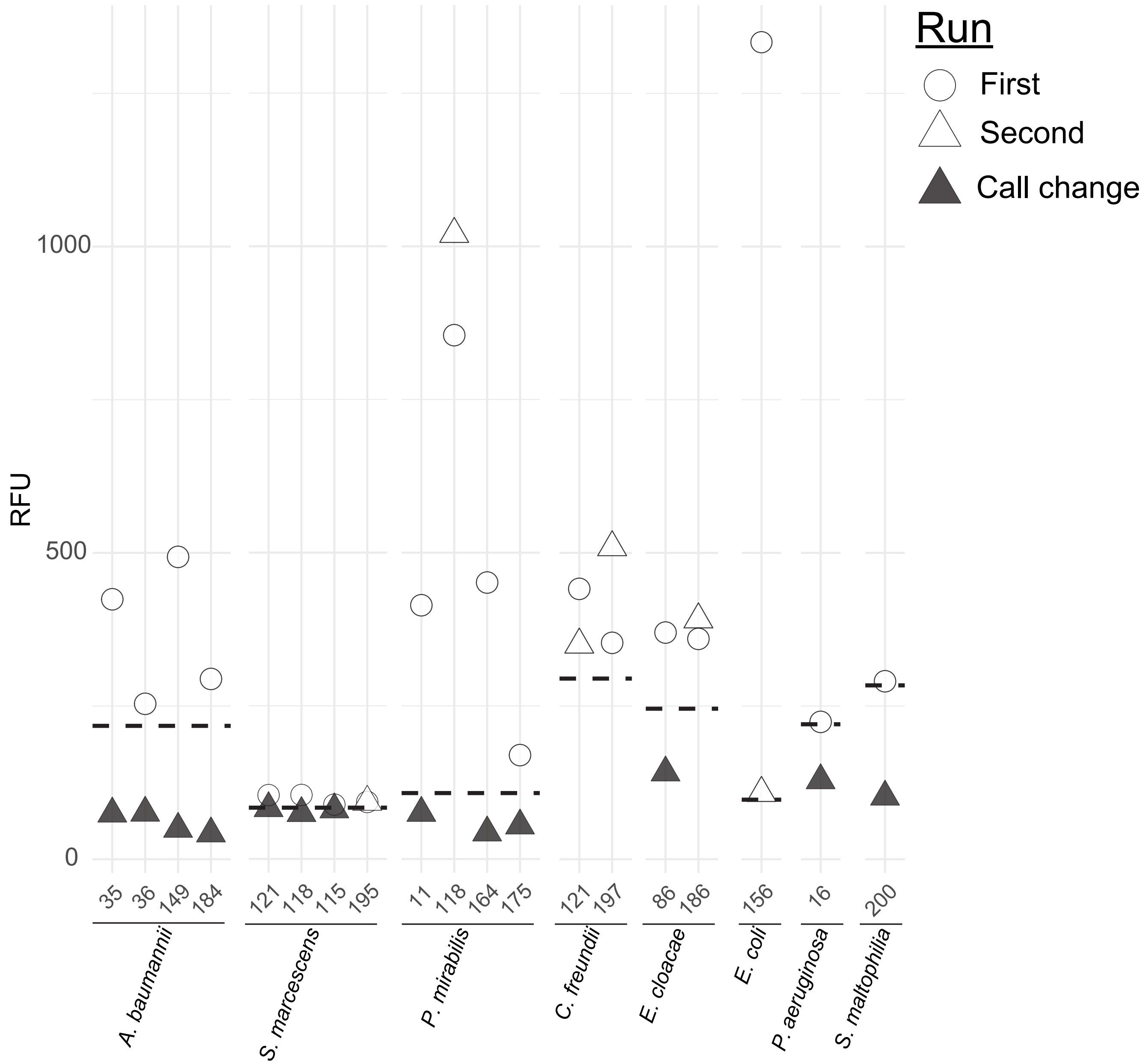

Figure S7. Repeat testing of discordantly positive samples in the BADLOCK assay. Each point represents a clinical sample that was initially positive by BADLOCK for the indicated species, which was not found by the reference method. For simplicity, only the discordant targets are shown. Samples are displayed on the x-axis and grouped by species, with shape distinguishing original versus repeat reactions. The dashed horizontal line indicates the species-specific RFU positivity threshold. A change in result classification occurs when a point crosses this threshold between reactions, marked by a shaded triangle. Upon retesting, 13 samples were corrected, whereas 6 remained discordant. Of these, two samples (IDs 195 and 156) yielded RFU values within 11% of the threshold, whereas the remaining four consistently showed strong positive signals above threshold.

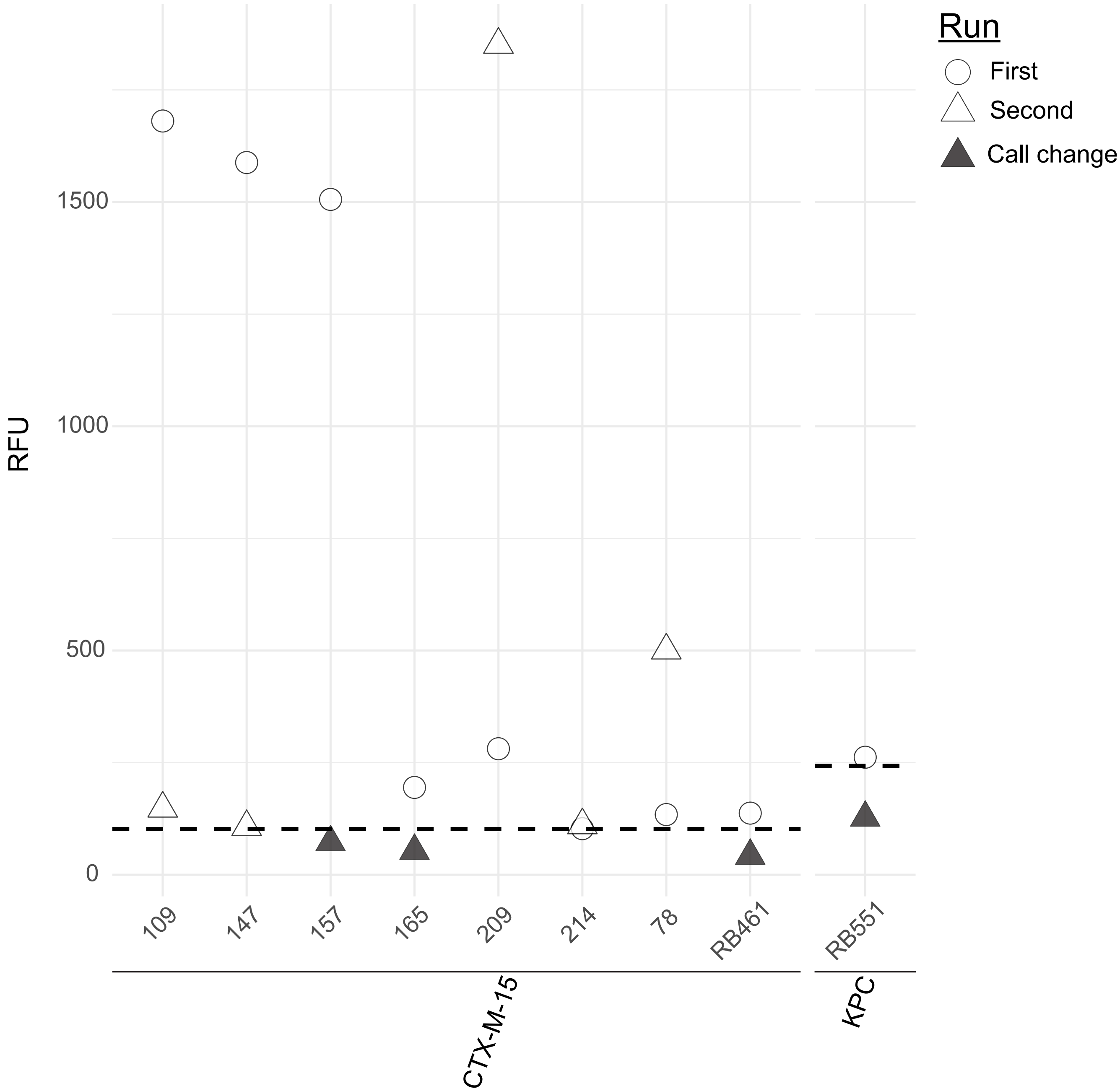

Figure S8. Repeat testing of discordantly positive samples in the BADLOCK AMR assay. Samples are displayed on the x-axis and grouped by AMR target, with shape distinguishing original versus repeat reactions. The dashed horizontal line indicates the target-specific RFU positivity threshold. A change in result classification occurs when a point crosses this threshold between reactions, marked by a shaded triangle.

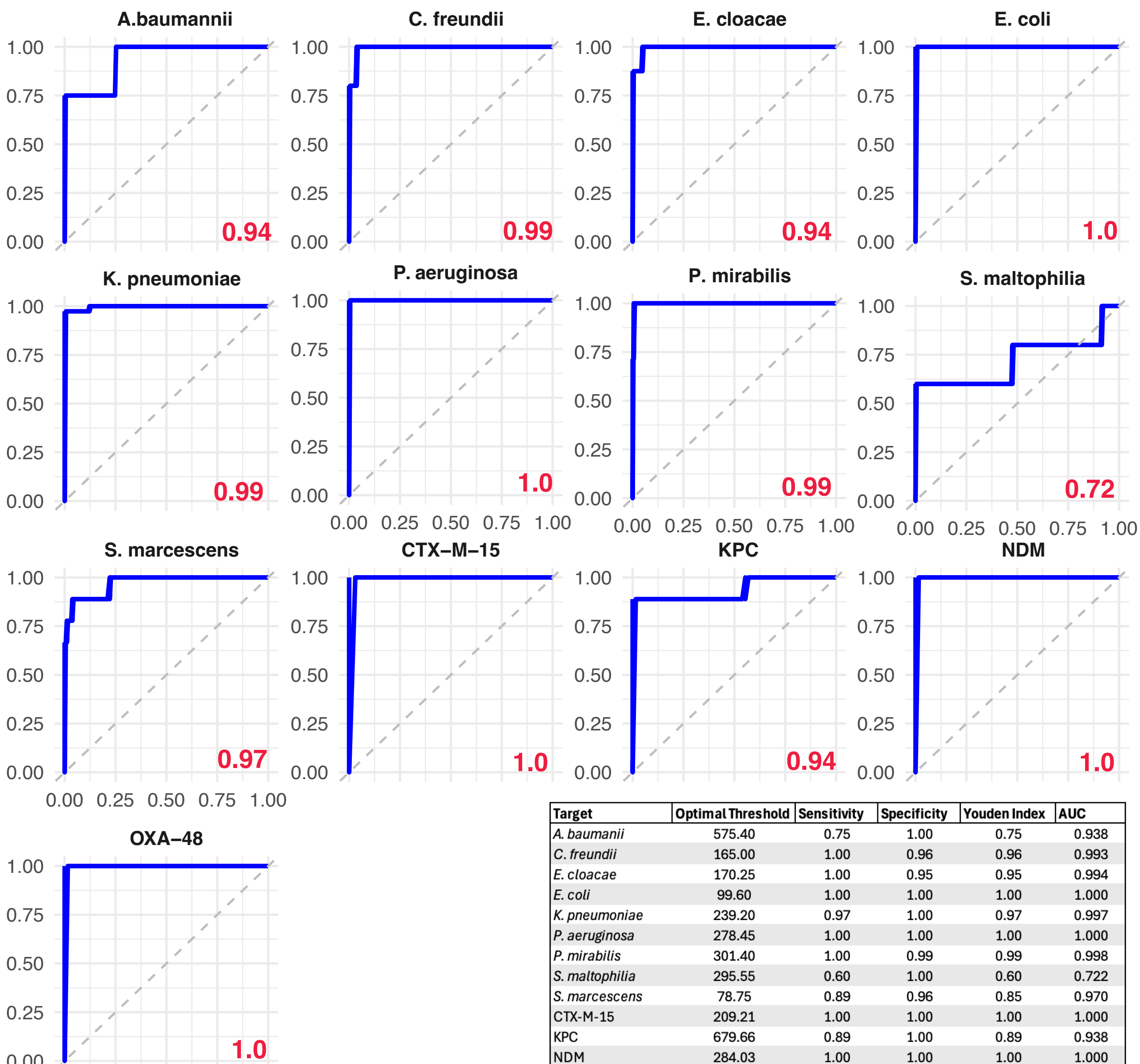

Target	Optimal Threshold	Sensitivity	Specificity	Youden Index	AUC
A. baumanii	575.40	0.75	1.00	0.75	0.938
C. freundii	165.00	1.00	0.96	0.96	0.993
E. cloacae	170.25	1.00	0.95	0.95	0.994
E. coli	99.60	1.00	1.00	1.00	1.000
K. pneumoniae	239.20	0.97	1.00	0.97	0.997
P. aeruginosa	278.45	1.00	1.00	1.00	1.000
P. mirabilis	301.40	1.00	0.99	0.99	0.998
S. maltophilia	295.55	0.60	1.00	0.60	0.722
S. marcescens	78.75	0.89	0.96	0.85	0.970
CTX-M-15	209.21	1.00	1.00	1.00	1.000
KPC	679.66	0.89	1.00	0.89	0.938
NDM	284.03	1.00	1.00	1.00	1.000
OXA-48	1035.10	1.00	1.00	1.00	1.000

Figure S9. Receiver operating characteristic (ROC) curves for each species-specific and AMR gene target. Curves were generated by comparing RFU values to known true-positive and true-negative classifications after excluding suspected contaminants (which we defined as samples with repeat RFU values >3-fold lower than the original) and *E. coli* false negatives lacking the *chuA* gene as confirmed by PCR. Each curve reflects the performance of a primer-guide target, with the area under the curve (AUC) in red text. Most targets demonstrated high discriminative ability, with AUC values ≥ 0.97 for the majority of species and AMR genes. The inset table displays the optimal threshold values along with their corresponding performance metrics across the cohort.

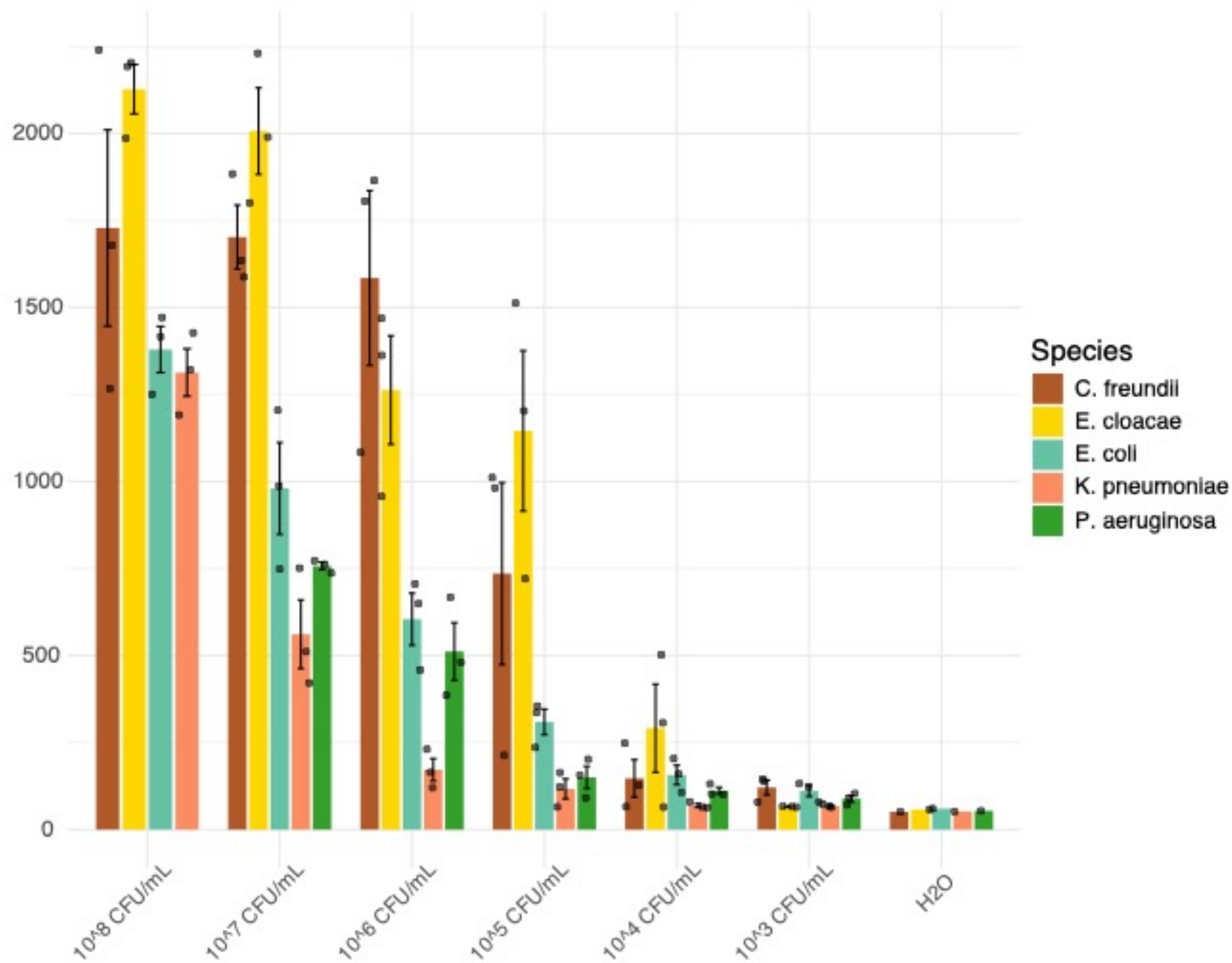

Figure S10. Limit of detection (LoD) for selected bacterial species included in the urine-based BADLOCK testing. Bars represent RFU values (y-axis) at each indicated cell density (x-axis). LoD values were determined using dilution series in pooled human urine, with detection defined as consistent assay positivity across replicates, as defined by target-specific thresholds in Fig. S11.

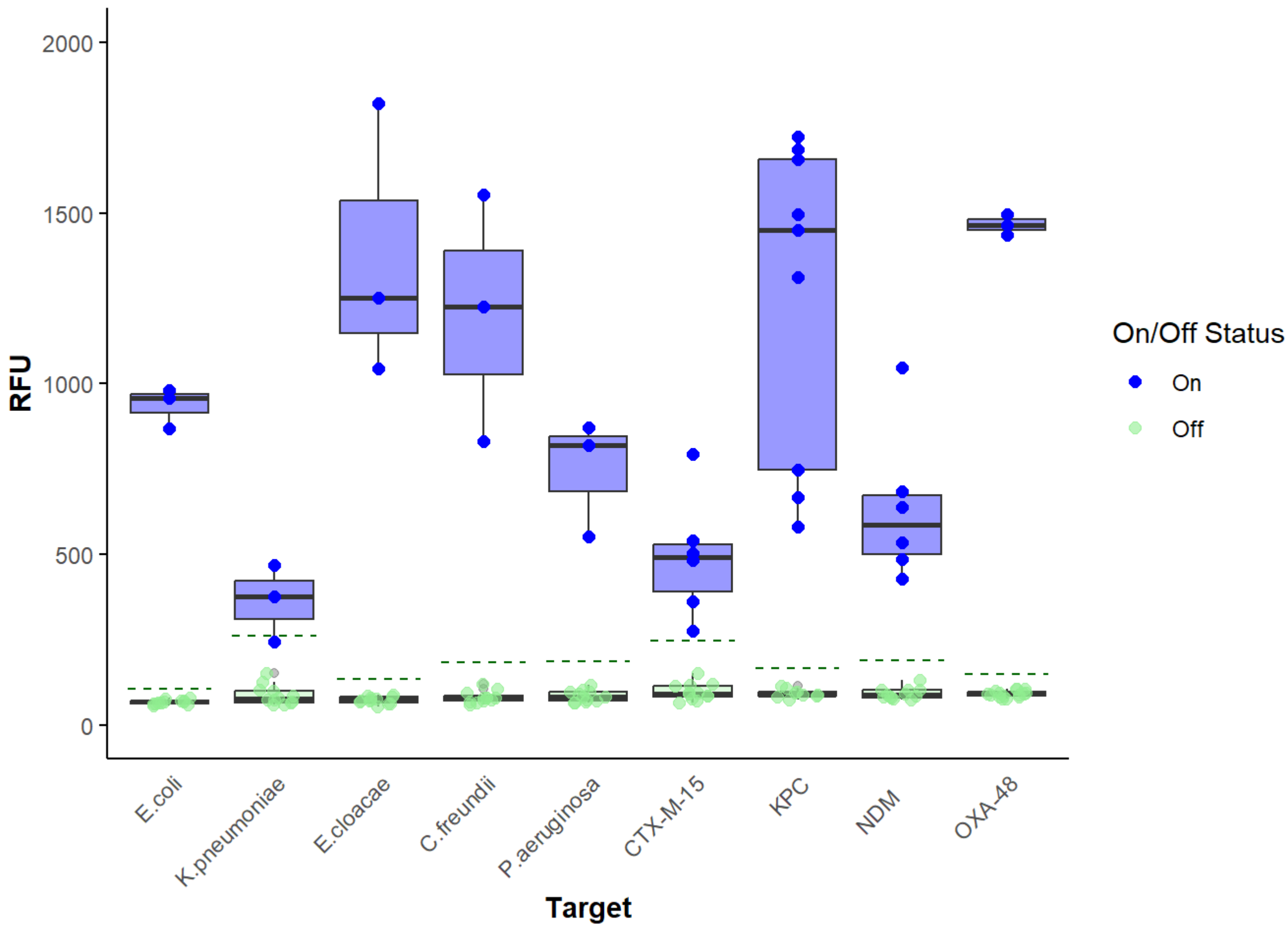

Figure S11. Threshold determination for species and antimicrobial resistance gene targets in the BADLOCK assay on commercial pooled human urine samples spiked with the indicated pathogen at 10^5 cfu/mL. Fluorescence values from technical replicates are shown. The horizontal dashed line indicates the threshold for calling a positive result, defined as the mean plus six times the standard deviation of fluorescence values from 12 off-target samples for species reactions and between 9 and 15 off-target samples for AMR gene reactions (variation due to some strains containing multiple target genes).

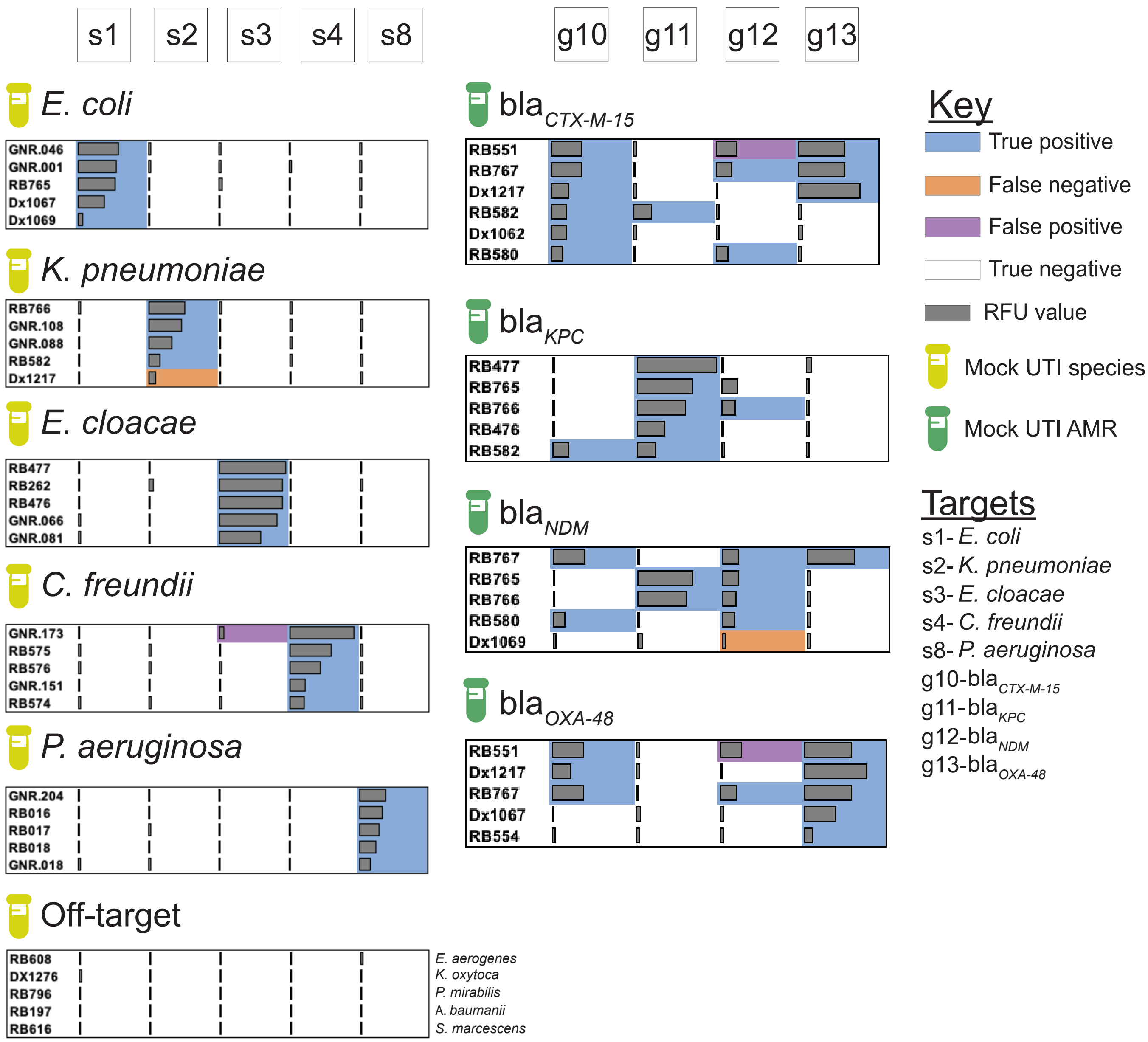

Figure S12. Consolidated visualization of BADLOCK assay runs for mock urinary tract infection (UTI) samples, including both species detection and AMR gene testing. Each row represents an individual sample, and each column corresponds to a BADLOCK target with the assay layout shown in the boxes above and listed in the accompanying key. Samples are grouped according to the species or AMR gene known to be present, and within each group, ordered by RFU of the on-target reaction. Each cell in the grid reflects a single reaction for a given sample, color-coded by call (see Key), with overlaid grey bars representing the corresponding RFU values. Symbols adjacent to each target indicate the cohort or target class (species or resistance gene), harmonized with the legend in main-text Figure 3. Samples containing multiple targets are shown in each group for which a corresponding target was present. The off-target species are listed next to the sample row.

Species	Target Gene	RPA Forward	RPA Reverse	Guide
E. coli	*chuA*	gaaattaatacgactcactatagggATATGGCGGTGAGTATTATCGTCAGGAACAACATC	GAGATGACCATTTGTCGGCATCAACATCTTTGTAG	CGGCGCGACGACGGGCTTCCCGCAAGCAgttttagtccccttcgtttttggggtagtctaaatcccctatagtgagtcgtattaatttc
K. pneumoniae	*topA*	gaaattaatacgactcactatagggGGTGACGCCCTGCCGCTGCAGGTAACCCATAA	ATCTTCACGCTCCAGTACGCTGTAGCTGGCTTT	GTGATGAGACCATGGCGGCGGTATCGCTgttttagtccccttcgtttttggggtagtctaaatcccctatagtgagtcgtattaatttc
P. aeruginosa	*lasB*	gaaattaatacgactcactatagggATGTTCTATCCGCTGGTGTCGCTGGA	ACCGCTGCCCTTCTTGATGTCGTAG	TTCTATATGCGCGGCAAGAACGACTTCCgttttagtccccttcgtttttggggtagtctaaatcccctatagtgagtcgtattaatttc
E. cloacae	*contig23*	gaaattaatacgactcactatagggAATGGGCGTATGATTATACCCAGGTAA	AGCAGTTTGGCAAAGTGCGCTTGTGCAG	AGTCAACTATTAGGCCAAAGCTATGATCgttttagtccccttcgtttttggggtagtctaaatcccctatagtgagtcgtattaatttc
C. freundii	*topA*	gaaattaatacgactcactatagggGTTGAAAACCGTCGCTTTTACGCTGAAAAA	TCCATTCGGCTTCTTTATTCGCCACCTGGTC	ATGGGTGAAATCGTCACCGATCGTCTGGgttttagtccccttcgtttttggggtagtctaaatcccctatagtgagtcgtattaatttc
S. maltophilia	*400kb_contig*	gaaattaatacgactcactatagggGACCATCCTGTACGCGCTGGGCTGGACGCAGC	CACCACGATTTCATCAGGCTGACGTGGAACTTC	TGTCCGACCTGCTGCCCGGCTACCTGACgttttagtccccttcgtttttggggtagtctaaatcccctatagtgagtcgtattaatttc
P. mirabilis	*topA*	gaaattaatacgactcactatagggCGGTACGCCTACTCGTCGAGCGTGAACGTG	CGTCACTTCCATACGCAAGGGTTGTTCATC	ATTGGCAATTACACGCTTCACTGCTGACgttttagtccccttcgtttttggggtagtctaaatcccctatagtgagtcgtattaatttc
A. baumannii	*topA*	gaaattaatacgactcactatagggGGCACTTACGCGAAGTGATTGGTGGTGAC	CACAGTAATGGCGAAACCATGAAGCCTAC	CAGCCAACACGTCTCGACTTAAACCGTGgttttagtccccttcgtttttggggtagtctaaatcccctatagtgagtcgtattaatttc
S. marcescens	*luxS*	gaaattaatacgactcactatagggGAAGAGCAGCGCGTTGCCGATGCCTGGAAAG	CTTTCGGCAGCGCCAGTTCGTCGTTGTGGT	TGTGGTACCTACCACATGCACTCGCTGGgttttagtccccttcgtttttggggtagtctaaatcccctatagtgagtcgtattaatttc
NA	KPC	gaaattaatacgactcactatagggCGTCTAGTTCTGCTGTCTTGTCTCTCATGG	CAAAGTCCTGTTCGAGTTTAGCGAATGGTT	GCTGGCTGGCTTTTCTGCCACCGCGCTGgttttagtccccttcgtttttggggtagtctaaatcccctatagtgagtcgtattaatttc
NA	NDM	gaaattaatacgactcactatagggAATGTCTGGCAGCACACTTCCTATCTCGAC	TGATCTCCTGCTTGATCCAGTTGAGGATCT	CAACGGTTTGATCGTCAGGGATGGCGGCgttttagtccccttcgtttttggggtagtctaaatcccctatagtgagtcgtattaatttc
NA	CTX-M-15	gaaattaatacgactcactatagggATAAAACCGGCAGCGGTGGCTATGG	GCTAATACATCGCGACGGCTTTCTGCCTTA	ATCGTGCGCCGCTGATTCTGGTCACTTAgttttagtccccttcgtttttggggtagtctaaatcccctatagtgagtcgtattaatttc
NA	OXA-48	gaaattaatacgactcactatagggTTAAAATTCCCAATAGCTTGATCGCCCTCG	ATAAACAGGCACAACTGAATATTTCATCGC	CCAAGTCTTTAAGTGGGATGGACAGACGgttttagtccccttcgtttttggggtagtctaaatcccctatagtgagtcgtattaatttc
Synthetic target	NA	gaaattaatacgactcactatagggaggg	TAGTTATTGCTCAGCGGTGG	GGCCGTCCGTTAATTTCCCTTGCATACAgttttagtccccttcgtttttggggtagtctaaatcccctatagtgagtcgtattaatttc

Table S1. Primer and guide sequences for each target gene included in our panel. Lowercase letters indicate conserved sequences (T7 primer sequence for the forward RPA primer, and non-targeting region of the guide sequence, respectively), whereas uppercase are the target-specific component.

Species	True (+)	False (+)	True (-)	False (-)	Sensitivity	Specificity	PPA	NPA
E.coli	66	1	115	12	0.85	0.99	0.85	0.99
K. pneumoniae	36	0	155	3	0.92	1.00	0.92	1.00
E. cloacae	7	2	184	1	0.88	0.99	0.88	0.99
C. freundii	4	2	187	1	0.80	0.99	0.80	0.99
S. marcescens	7	4	181	2	0.78	0.98	0.78	0.98
P. mirabilis	7	4	183	0	1.00	0.98	1.00	0.98
S. maltophilia	3	1	188	2	0.60	0.99	0.60	0.99
P. aeruginosa	8	1	185	0	1.00	0.99	1.00	0.99
A. baumannii	3	4	186	1	0.75	0.98	0.75	0.98

Table S2. Performance characteristics for each species target. Note that 11 of the 12 false negative *E. coli* samples lacked the *chuA* target gene.

Sample ID	Published ID	Source of Strain	Cohorts	Species	AMR genes
DX1003	MGH145	PMID: 28096418	AMR	*C. freundii*	KPC-2
DX1034	DH10Beta + pBAD_KPC3	Bhattacharyya Lab	AMR	*E. coli*	KPC-3
DX1062	RB001+CTX-M-15	Bhattacharyya Lab	AMR, Urine species	*E. coli*	CTX-M-15
DX1067	RB001+OXA-48	Bhattacharyya Lab	AMR, Urine species, Urine AMR	*E. coli*	OXA-48
DX1068	RB059+OXA-48	Bhattacharyya Lab	AMR	*E. coli*	OXA-48
DX1069	RB001+NDM-1	Bhattacharyya Lab	AMR, Urine species, Urine AMR	*E. coli*	NDM-1
DX1217	AR0066 (CDC)	CDC AR Isolate Bank	AMR, Urine species, Urine AMR	*K. pneumoniae*	CTX-M-15, OXA-232
DX1218	AR0068 (CDC)	CDC AR Isolate Bank	AMR	*K. pneumoniae*	CTX-M-15, NDM-1, OXA-48
DX1219	AR0143 (CDC)	CDC AR Isolate Bank	AMR	*K. pneumoniae*	CTX-M-15, NDM-1
DX1220	AR0153 (CDC)	CDC AR Isolate Bank	AMR	*K. pneumoniae*	CTX-M-15, NDM-1, OXA-48
DX1276	MGH175	PMID: 28096418	Urine species	*K. oxytoca*	na
RB016	2732233	Bhattacharyya Lab	Urine species	*P.aeruginosa*	na
RB017	B895023	Bhattacharyya Lab	Urine species	*P.aeruginosa*	na
RB018	2762085	Bhattacharyya Lab	Urine species	*P.aeruginosa*	na
RB197	Acinetobacter #1	Bhattacharyya Lab	Urine species	*A. baumannii*	na
RB262	SBJ-32413; 1349	Bhattacharyya Lab	Urine species	*E. cloacae*	na
RB459	BIDMC68	PMID: 28096418	AMR	*K. pneumoniae*	KPC-2
RB461	MGH67	PMID: 28096418	AMR	*K. pneumoniae*	KPC-2
RB462	MGH71	PMID: 28096418	AMR	*K. pneumoniae*	KPC-2
RB476	BWH39	PMID: 28096418	AMR, Urine species, Urine AMR	*E. cloacae*	KPC-2
RB477	MGH5	PMID: 28096418	AMR, Urine species, Urine AMR	*E. cloacae*	KPC-2
RB551	BWH2	PMID: 28096418	AMR, Urine AMR	*K. pneumoniae*	CTX-M-15, OXA-48
RB554	BAA2524	PMID: 28096418	AMR, Urine AMR	*K. pneumoniae*	OXA-48
RB574	MGH281c	PMID: 28096418	Urine species	*C. freundii*	na
RB575	MGH281	PMID: 28096418	Urine species	*C. freundii*	na
RB576	MGH283	PMID: 28096418	Urine species	*C. freundii*	na
RB579	IDR1600031102-01-00	Wadsworth Lab	AMR	*K. pneumoniae*	CTX-M-15
RB580	IDR1600014966-01-00	Wadsworth Lab	AMR, Urine AMR	*Citrobacter spp*	CTX-M-15, NDM-1
RB582	IDR1600037319-01-00	Wadsworth Lab	AMR, Urine species, Urine AMR	*K. pneumoniae*	CTX-M-15, KPC-2
RB608	BAC0800002538	Wadsworth Lab	Urine species	*E. aerogenes*	na
RB616	IDR1300010999	Wadsworth Lab	Urine species	*S. marcescens*	na
RB765	F3461517	PMID: 28096418	AMR, Urine species, Urine AMR	*E. coli*	KPC-2, NDM-1
RB766	F3321027	PMID: 28096418	AMR, Urine species, Urine AMR	*K. pneumoniae*	KPC-2, NDM-1
RB767	X1621267	PMID: 28096418	AMR, Urine AMR	*E. coli*	CTX-M-15, NDM-1, OXA-48
RB796	MGH013	PMID: 28096418	Urine species	*P. mirabilis*	na

Table S3. Banked clinical isolates with known genomic content. The table includes the testing cohort(s) in which each sample was used, the species name, and the relevant antimicrobial resistance gene(s) of interest.

Supplementary Data File: A Supplementary Data File, linked from the manuscript, includes raw data from fluorescence measurements (relative fluorescence units, RFU) from BADLOCK reactions that underlie all main figures in the manuscript. All data are presented as barplots but encoded in each cell as raw RFU values. For each value, cell background color indicates True Positive (blue), True Negative (white), False Positive (purple), or False Negative (orange), based on comparisons to a threshold value for detection (set as described in the manuscript), and the identification in the clinical microbiology laboratory (for clinical samples), or whole genome sequencing or PCR (for laboratory isolates). As described in each tab of the Excel workbook, contents include:

- Tab 1: RFU data from clinical samples tested with the BADLOCK GNR species panel, grouped by species identified by clinical microbiology workflow as indicated in column A. Data from polymicrobial samples are duplicated such that they are listed in each relevant group (e.g., sample 42 contains both *E. coli* and *K. pneumoniae*, thus RFU data from the same run are duplicated in both the *E. coli* and the *K. pneumoniae* groups). Within each group, samples are ordered by RFU of the matching probe. For the last group, representing samples with exclusively one or more off-panel organisms, the species or genus identified by standard clinical microbiology laboratory workflow is indicated at right.
- Tab 2: RFU data from clinical samples tested with the BADLOCK AMR gene panel, ordered by RFU of the CTX-M-15 probe (the most common on-panel gene present). Known gene content (verified as described in the manuscript) is listed in column A.
- Tab 3: RFU data from banked laboratory isolates tested with the BADLOCK AMR gene panel, grouped by AMR gene as indicated in column A. Data from isolates with multiple AMR genes are duplicated in each group (e.g., isolate RB582 encodes both $\textit{bla}_{\text{CTX-M-15}}$ and $\textit{bla}_{\text{KPC}}$, thus RFU data from the same run are duplicated in both the CTX-M-15 and the KPC group).
- Tab 4: RFU data from mock urine samples, generated as described in the manuscript, tested with the BADLOCK urine GNR species panel (a subset of the full GNR species panel), grouped by known species as indicated in column A. Within each group, samples are ordered by RFU of the matching probe. For the last group, representing off-panel species, the species or genus is indicated at right.
- Tab 5: RFU data from mock urine samples, generated as described in the manuscript, tested with the BADLOCK AMR gene panel, grouped by AMR gene as indicated in column A. Data from isolates with multiple AMR genes are duplicated in each group, as in Tab 3.
